# Challenges of neuropathic pain: focus on diabetic neuropathy

**DOI:** 10.1007/s00702-020-02145-7

**Published:** 2020-02-08

**Authors:** Daniela C. Rosenberger, Vivian Blechschmidt, Hans Timmerman, André Wolff, Rolf-Detlef Treede

**Affiliations:** 1 Department of Neurophysiology, Mannheim Center for Translational Neuroscience (MCTN), Medical Faculty Mannheim, University of Heidelberg, Heidelberg, Germany; 2 Department of Anesthesiology, Pain Center, University Medical Center of Groningen (UMCG), University of Groningen, Groningen, The Netherlands

**Keywords:** Painful diabetic neuropathy, Spinal sensitization, Neuroinflammation, Quantitative sensory testing, Stratification in clinical trials, Personalized pain management

## Abstract

Neuropathic pain is a frequent condition caused by a lesion or disease of the central or peripheral somatosensory nervous system. A frequent cause of peripheral neuropathic pain is diabetic neuropathy. Its complex pathophysiology is not yet fully elucidated, which contributes to underassessment and undertreatment. A mechanism-based treatment of painful diabetic neuropathy is challenging but phenotype-based stratification might be a way to develop individualized therapeutic concepts. Our goal is to review current knowledge of the pathophysiology of peripheral neuropathic pain, particularly painful diabetic neuropathy. We discuss state-of-the-art clinical assessment, validity of diagnostic and screening tools, and recommendations for the management of diabetic neuropathic pain including approaches towards personalized pain management. We also propose a research agenda for translational research including patient stratification for clinical trials and improved preclinical models in relation to current knowledge of underlying mechanisms.

## Introduction

Numerous reviews have been written about neuropathic pain (NP) in general (see, e.g., Baron 2006; Campbell and Meyer 2006; Colloca et al. 2017; Meacham et al. 2017) and painful diabetic neuropathy (pDN) in particular (see, e.g., Feldman et al. 2019; Nawroth et al. 2018; Sloan et al. 2018). Many of them gave insight into recent findings on mechanisms of NP that may help to understand and further develop strategies for correct diagnosis and successful treatment. Although screening and diagnostic tools have become more and more available (Haanpaa et al. 2011), NP is considered to be an underdiagnosed condition because a clear, comprehensive classification has been lacking until recently (Finnerup et al. 2013). NP is no longer called “chronic intractable pain”, but its management remains difficult: with current pharmacologic concepts that are internationally recommended by guidelines, only 30% of patients experience a pain reduction of about 30% (Finnerup et al. 2015). The aim of this paper is to review mechanisms, assessment, classification, and management of peripheral NP. We will also discuss to what extent these underlying mechanisms have been considered in the development of diagnostic or treatment strategies in patients with painful (pDN) and painless diabetic polyneuropathy (dPNP) and what has proven to be useful. Given the importance as a global burden and rising number in patients as one of the main causes of NP (Rice et al. 2016; IDF Diabetes Atlas; van Hecke et al. 2014), the main focus will be on pDN due to its high and increasing prevalence.

### Definitions

According to the taxonomy of the International Association for the Study of Pain (IASP 2011; Loeser and Treede 2008), neuropathic pain (NP) is defined as “*pain caused by a lesion or disease of the somatosensory nervous system*”. The definite diagnosis of NP requires a demonstrable underlying lesion or disease satisfying established neurological diagnostic criteria (Finnerup et al. 2016; Loeser and Treede 2008; Treede et al. 2008). Painful diabetic neuropathy (pDN) is a frequent subtype of peripheral NP; it is defined as “*pain as a direct consequence of abnormalities in the peripheral somatosensory system in people with diabetes*” (Jensen et al. 2011; Tesfaye et al. 2010).

IASP taxonomy differentiates NP from nociceptive pain and—more recently—nociplastic pain. Nociceptive pain describes “*pain through activation of nociceptors in non-neural tissues by actual or threatened tissue injury*”, while nociplastic pain is defined as “*pain that arises from altered nociception despite no clear evidence of actual or threatened tissue damage causing the activation of peripheral nociceptors or evidence for disease or lesion of the somatosensory system causing the pain*” (IASP 2011; Kosek et al. 2016; Loeser and Treede 2008). This distinction is essential, as different underlying mechanisms explain different treatment targets and responses to drugs. However, patients may present a substantial overlap of neuropathic and nociceptive pain in the same areas, e.g., in low back pain, postsurgical pain or osteoarthritis; this overlap has been called “mixed pain” (Freynhagen et al. 2019). Patients with substantial overlap of neuropathic and nociplastic pain are likely to exist also, but there are no systematic studies yet.

### Classification of neuropathic pain

Neuropathic pain may be classified according to the underlying lesion or disease (Scholz et al. 2019) or according to the clinical phenotype (Vollert et al. 2018). While the clinical phenotype may be useful for future personalized NP management (see below), the 11th edition of the International Classification of Diseases (ICD-11) differentiates NP of peripheral and central origin, comprising nine typical conditions associated with persistent or recurrent pain (Scholz et al. 2019, Table 1). There are also extension codes for pain severity (combining intensity, distress, and disability), temporal characteristics and psychological or social factors, as well as a link to the International Classification of Functioning (ICF) (Scholz et al. 2019; Treede et al. 2019; Nugraha et al. 2019; WHO Classification 2001). Generally, NP is considered to be chronic, as it either persists continuously or manifests with recurrent painful episodes and is usually not limited by the natural healing process or treatment of the underlying disease. The IASP classification of chronic NP for ICD-11 represents the first systematic classification to date of common painful neurological disorders; member states are expected to report health statistics to WHO according to ICD-11 from 2022 onward. Thus, pDN is classified as *chronic NP* (top /first-level diagnosis) of peripheral origin (*chronic peripheral NP*; second-level diagnosis), *painful polyneuropathy* (third-level diagnosis) (Scholz et al. 2019). From the clinical point of view, a physical examination is crucial to (1) link the patient’s pain to a lesion or disease of the somatosensory nervous system, (2) to distinguish the NP component from nociceptive pain, and (3) to distinguish the NP component from nociplastic pain.

**Table 1:** Classification of chronic neuropathic pain in ICD-11

*Top/first-level diagnosis*	
Chronic neuropathic pain	
*Second-level diagnosis*	
Chronic peripheral NP	Chronic central NP
*Third-level diagnosis*	
Trigeminal neuralgia^a^ Chronic NP after peripheral nerve injury^a^ Painful polyneuropathy Postherpetic neuralgia Painful radiculopathy	Chronic central NP associated with spinal cord injury^a^ Chronic central NP associated with brain injury^a^ Chronic central post-stroke pain Chronic central NP associated with MS

According to Scholz et al. (2019)

^a^ICD-11 introduces the concept of multiple parenting, i.e., certain diagnoses may be listed in other divisions of the chronic pain classification, too, such as chronic posttraumatic pain or orofacial pain. Here, multiple parents are not listed for better readability

### Etiology

Neuropathic pain may result from a broad range of diverse neurological disorders affecting the peripheral or the central nervous system (Table 2). Chronic pain may also occur in neurological conditions of unknown etiology, i.e., idiopathic neuropathies (Colloca et al. 2017). However, not all patients affected by neural disorders or lesions do develop NP. Extent and severity of NP vary markedly between patients suffering from the same underlying disease or neural lesions, particularly in diabetic polyneuropathy (dPNP) (Themistocleous et al. 2016). Whether or not patients develop NP seems to be a multifactorial interaction of psychosocial, genetic, biological, and clinical risk factors (Hebert et al. 2017). A large (~ 10,000 participants), currently running multi-center observational study, DOLORisk, aims to elucidate these risk factors of development of NP (Pascal et al. 2018).

**Table 2:** Neuropathic pain due to peripheral nerve damage

Etiology	Typical syndromes (examples)	Experimental models
Mechanical (compressive/traumatic)	Carpal tunnel syndrome Postsurgical pain Painful radiculopathy Cancer pain Phantom limb pain	Complete or partial nerve transection, chronic constriction or compression of peripheral nerves
Metabolic/ischemic	Diabetic polyneuropathy Vitamin B12 deficiency	dPNP: hyperglycemic condition or streptozotocin induced; genetic models
Inflammatory (infectious/autoimmune)	Post-herpetic neuralgia HIV neuropathy Leprosy Guillain–Barré Syndrome Critical illness polyneuropathy	Injection of viral proteins or cells systemically or specifically to e.g., sciatic nerve Rat sepsis model^a^
Toxic	Chemotherapy-induced peripheral neuropathy Alcoholic neuropathy	Injection of drugs or ethanol, systemically or specifically to, e.g., sciatic nerve
Radiation	Post-radiation neuropathy	X-radiation on peripheral nerves of the mouse^d^
Hereditary	Charcot–Marie–Tooth disease Fabry disease	Genetic model (e.g., α-GAL-deficient mice for Fabry disease)

Typical neuropathic pain syndromes^b^ and corresponding experimental animal models^c^, sorted according to mechanisms of peripheral nerve damage (etiologies)

*dPNP* diabetic polyneuropathy

^a^Nardelli et al. (2013)

^b^For a very detailed overview of possible causes of NP, see review by Jay and Barkin (2014)

^c^For more details on animal models of NP in general, see Jaggi et al. (2011), Gregory et al. (2013), and Challa (2015). For animal models particularly on dPNP, see Gao and Zheng (2014)

^d^Love (1983) and Jiang et al. (2017)

### Epidemiology

Chronic NP frequently causes major suffering, a reduced quality of life and disability in patients, and is a major factor contributing to the global burden of disease (Doth et al. 2010; Smith and Torrance 2012; Alleman et al. 2015; Rice et al. 2016). For the general population, a prevalence of NP of 6.9–10% is estimated (Bouhassira et al. 2008; Attal et al. 2018). The prevalence of NP is likely to increase as we are facing, among other risk factors, an aging population, increasing obesity rates and an increase in survival of cancer patients that may suffer from sequelae of chemotherapeutics (Moulin et al. 2014). However, systematic registration of incidence and prevalence of NP in the general population is difficult because the current versions of the International Classification of Disease (ICD-9 or ICD-10) are focused on the underlying lesions or diseases and not on whether or not they are painful (Finnerup et al. 2013). Such data have only been obtained by dedicated surveys in certain countries or for certain etiologies (Colloca et al. 2017). Generally, the association of pain and the underlying neurological disease is highly variable. While in some diseases such as postherpetic neuralgia or trigeminal neuralgia, pain is the most prominent manifestation, in others such as chemotherapy-induced neuropathy or dPNP, it may occur only in a subgroup of patients (Table 3). Even among patients with the same underlying cause of NP, painful symptoms and signs may differ depending on the studied population, the diagnostic tools or criteria (Nawroth et al. 2018).

**Table 3:** Prevalence of neuropathic pain in the general population and in common underlying diseases

General population	6.9 to 10%	Bouhassira et al. (2008), Colloca et al. (2017), Attal et al. (2018)
*Central neuropathic pain*		
Spinal cord injury	53 to 85%	Burke et al. (2017), Hatch et al. (2018)
Stroke	8 to 30%	Delpont et al. (2018)
Multiple sclerosis	29%	Foley et al. (2013)
*Peripheral neuropathic pain*		
Herpes zoster	5 to 67%	Mallick-Searle et al. (2016), Forbes et al. (2016)
Postherpetic neuralgia^a^	100% per definition	
Diabetes mellitus	~ 20 to 50%	Alleman et al. (2015), Sloan et al. (2018)
HIV neuropathy	~ 20%	Ellis et al. (2010)
Trigeminal neuralgia^a^	100% per definition	
Post amputation	60%	Manchikanti and Singh (2004)
Post-surgical	10–50%	Borsook et al. (2013)

Most references are specific systematic literature reviews. Some did include questionnaire-based screening for the assessment of NP or telephone interviews for follow-up. Ellis et al. (2010) is about the CHARTER study, a longitudinal cohort study

^a^These diseases are neuropathic pain conditions according to their clinical definition

Given the increasing prevalence of diabetes mellitus (DM) worldwide, dPNP is and will be one of the most important and common causes of NP. In 2000, 171 million (2.8% of the world population) people suffered from DM (Wild et al. 2004), projections at the time for 2030 of 366 million (4.4%) are already by far surpassed. Today, in 2019, 425 million (8.6%) are affected; in 2045 629 million (9.8%) people are expected with DM worldwide (IDF Diabetes Atlas; United Nations (2019) Revision of World Population Prospects). dPNP is a frequent complication of long-term diabetes and one of the leading causes of morbidity and disability. While up to 60% in patients with chronic DM are affected by dPNP, already in newly diagnosed patients, 7–10% suffer from neuropathy (Tracy and Dyck 2008; Tesfaye 2010; Abbott 2011). It seems to be generally more prevalent in Europeans as compared with Asians (Abbott et al. 2005). In dPNP, NP is one of the main symptoms. Mostly, patients suffering from pDN are regarded as a subgroup of dPNP patients (≤ 60%, Abbott et al. 2011). However, in one-fourth of all DM patients, painful symptoms occur without any other signs of neuropathy (Abbott et al. 2011). Of all DM patients, 20–50% suffer from pDN (Abbott et al. 2011; Bouhassira et al. 2013; Alleman et al. 2015; Sloan et al. 2018; Truini et al. 2018).

The burden of disease in pDN is much higher than in other chronic pain conditions (Sadosky et al. 2015) resulting in reduced health-related quality of life (van Acker 2009; Callaghan et al. 2012a; Smith et al. 2012; Bouhassira et al. 2013; Alleman et al. 2015; Finnerup et al. 2015; Finnerup et al. 2016): comorbidities, such as sleep disorders, anxiety/depression (Gore et al. 2005; Jain et al. 2011) and cardiovascular diseases (Sadosky et al. 2015), and “severe” pain in more than half of the affected patients (Sadosky et al. 2015). Even 10-year mortality is higher in patients suffering from pDN than in patients without pain (Torrance et al. 2010).

## Pathophysiology of peripheral neuropathic pain

Neuropathic pain (NP) can be divided into central or peripheral syndromes, depending on the site of lesion or underlying disease. This section focuses on conditions that are considered consequences of a peripheral insult. Central NP conditions are less well understood and might differ in their underlying mechanisms, so they need separate consideration (Watson and Sandroni 2016).

Peripheral nerve damage provokes persistent maladaptive structural and functional responses in the somatosensory system. Therefore, peripheral NP results from both, peripheral and central mechanisms. Clinical signs include sensory loss, spontaneous (ongoing) pain and hypersensitivity, including allodynia and hyperalgesia (evoked pain) (Jensen and Finnerup 2014).

**Fig. 1:**
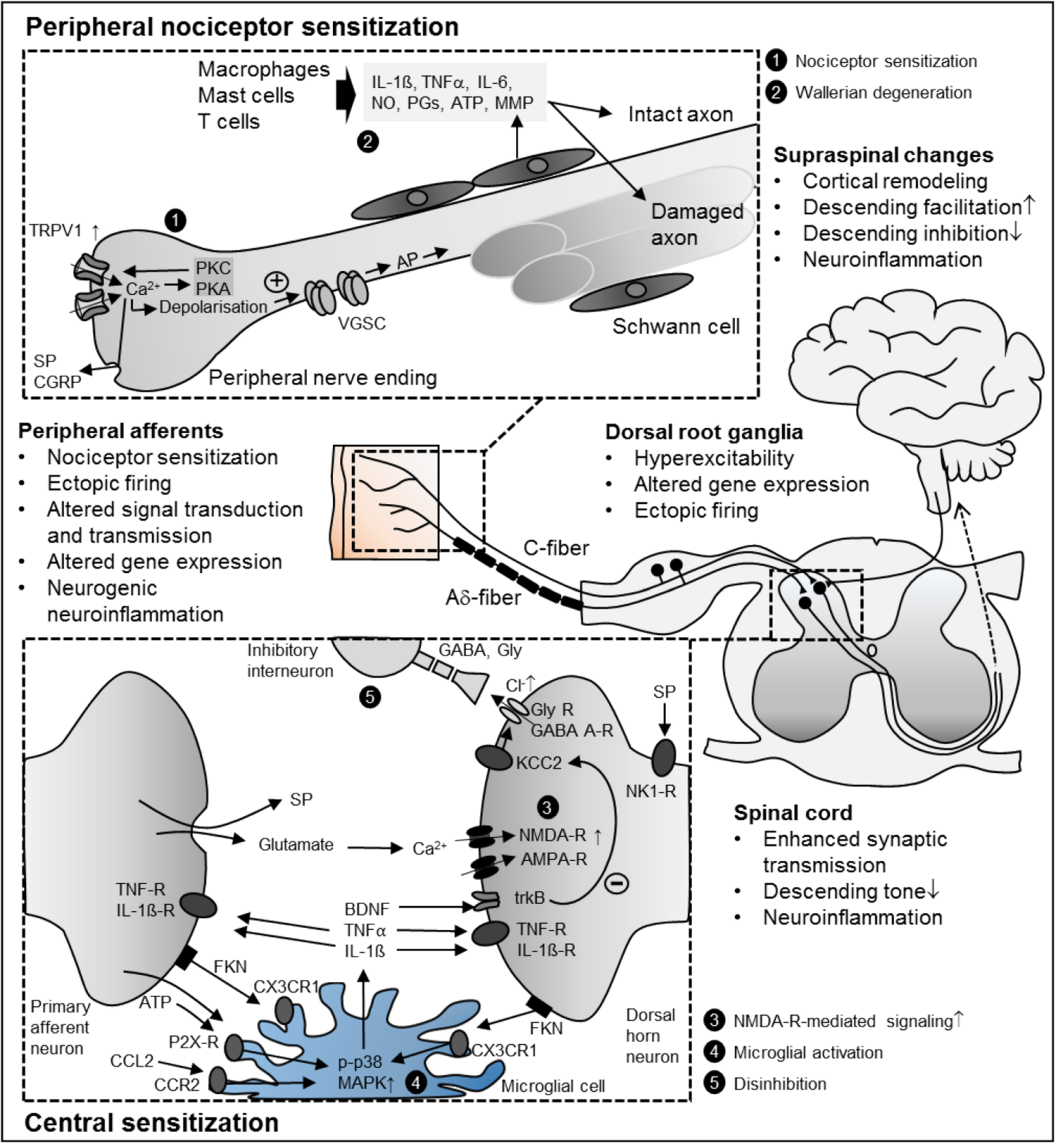
Selection of peripheral and central mechanisms contributing to neuropathic pain. *AMPA-R/NMDA-R* ionotropic glutamate receptors, *AP* action potential, *ATP* adenosine triphosphate, *BDNF* brain-derived neurotrophic factor, *CCL2/FKN* chemokines, *CCR2/CX3CR1* chemokine receptors, *CGRP* calcitonin gene-related peptide, *GABA* gamma-aminobutyric acid, *Gly* Glycin, *FKN* fractalkine (CX3CL1), *IL-1β* interleukin 1β, *IL-6* interleukin 6, *KCC2* chloride potassium symporter, *MMP* matrix metalloproteinase, *NK1-R* neurokinin 1 receptor, *NO* nitric oxide, *p-p38* MAPK phosphorylated p38 mitogen-activated protein kinase, *PG* prostaglandins, *SP* substance P, *TNFα* tumor necrosis factor-alpha, *TNF-R* tumor necrosis factor receptor, *trkB* tyrosine kinase B, *TRPV1* transient receptor potential vanilloid 1, *VGSC* voltage-gated sodium channel

Most of the current ideas regarding the pathophysiology of NP have been derived from animal models of mechanical nerve damage, such as spared nerve injury (SNI), chronic constriction injury (CCI), and spinal nerve ligation (SNL). Additionally, pathogenesis of NP has also been studied in rodent models of diabetes, chemotherapy, herpes zoster and HIV–peripheral neuropathy (Colleoni and Sacerdote 2010). These preclinical studies delineated a series of mechanisms along the entire nervous system (Fig. 1). In the peripheral nervous system (PNS), nerve damage leads to reduced signal transmission to the spinal cord and alterations in gene expression patterns and ion channel properties leading to ectopic activity. In the central nervous system (CNS), enhanced synaptic transmission and disinhibition at the spinal, thalamic and cortical level lead to amplified central processing. Human studies revealed some of these mechanisms in patients with NP and in human surrogate models of NP (Binder 2016; Klein et al. 2005; Vollert et al. 2018). In the following sections, a short overview of these mechanisms is given to understand current and future strategies for the assessment and treatment of NP.

### Mechanisms of sensory loss

After peripheral nerve injury, neurodegeneration disrupts the connection between the periphery and the CNS, ultimately resulting in sensory loss. After transection of axons of primary sensory neurons, the distal axons die due to Wallerian degeneration (Campbell and Meyer 2006), particularly affecting small-fiber neurons including nociceptors (Tandrup et al. 2000). Later on, persistent aberrant afferent input may provoke the degeneration of superficial dorsal horn neurons via glutamate-mediated excitotoxicity (Scholz et al. 2005). Neuroimaging studies in patients with NP hint that neurodegeneration may also occur in the brain (May 2008).

### Mechanisms of ongoing pain

Meanwhile, the proximal remnants of the fibers (e.g., C-fibers) at the injury site can generate ectopic activity and so pain originates from an area with reduced sensitivity to thermal and mechanical stimuli. Microneurographic recordings of single C-fibers have demonstrated spontaneous activity in human studies investigating several NP syndromes (Serra et al. 2012). Ongoing pain, such as burning ongoing pain and spontaneous shock-like pain, is the most prevalent feature and most troublesome clinical sign in NP syndromes (Gold and Gebhart 2010). Since ongoing pain can be temporarily abolished by blocking peripheral input, research focuses on the primary afferent fiber as the origin of ongoing pain (Gracely et al. 1992; Haroutounian et al. 2014). Ongoing pain is thought to result from ectopic action potential (AP) generation within the nociceptive pathways through enhanced synaptic transmission to the spinal neurons and/or enhanced intrinsic excitability of second-order neurons (Woolf et al. 1992; Balasubramanyan et al. 2006; Hains and Waxman 2007). Ectopic discharge was originally described as arising only at the site of the nerve lesion (Wall and Gutnick 1974), but can occur at multiple sites, including the site of injury, along the axon and in the dorsal root ganglia (DRG) of nociceptors (Devor 2009). Enhanced sensitivity of primary sensory neurons to endogenous thermal and chemical stimuli may also cause spontaneous pain.

Ectopic discharge is associated with increased expression of voltage-gated sodium channels (VGSC) in primary afferents (Cummins et al. 2007). Clustering of VGSC might lower the action potential (AP) threshold at sites of ectopic impulses resulting in hyperexcitability (Lai et al. 2003). In peripheral sensory neurons, the VGSC subtypes Nav1.7, Nav1.8, and Nav1.9 are particularly prevalent. Their contribution to pain pathogenesis varies in different NP conditions (Dib-Hajj et al. 2010; Hameed 2019). Rare inherited channelopathies show a crucial role of VGSC in pain processing (Bennett and Woods 2014; Hoeijmakers et al. 2015); loss-of-function mutations in Nav1.7 are associated with insensitivity to pain (Cox et al. 2006), while gain-of-function mutations in Nav1.7 lead to hyperexcitability and pain disorders in humans, erythromelalgia and paroxysmal extreme pain disorder (Estacion et al. 2008). Neurotrophic factors induce alterations in the VGSC, e.g., time-dependent changes in Nav1.8 (Amir et al. 2006; Coward et al. 2000), including upregulation, low excitability threshold and an increased suprathreshold ion current (Lai et al. 2004). Nav1.9 might also contribute to increased excitability in NP (Hoffmann et al. 2017). After nerve injury, large numbers of fast Nav1.3 are expressed, which otherwise are only present during embryonic development. Nav1.3 causes strong fluctuations of the membrane potential and is probably the cause of spontaneously arising AP bursts (Wood et al. 2004).

Some NP conditions, however, are independent of VGSC (Minett et al. 2014). Apart from VGSC, some types of calcium channels (Zamponi et al. 2009), potassium channels (Busserolles et al. 2016), and hyperpolarization-activated cyclic nucleotide-gated channels (Chaplan et al. 2003) also contribute to hyperexcitability.

### Peripheral nociceptor sensitization

An important characteristic of nociceptors, such as unmyelinated (C) and thinly myelinated (Aδ) primary afferent neurons, is sensitization. Sensitization, which typically develops as a consequence of tissue injury and inflammation, is defined as a reduction in the threshold, an increase in the magnitude of response to noxious stimulation and spontaneous activity. The inflammatory processes in Wallerian degeneration may hence render the remaining intact fibers after nerve injury hyperexcitable (Campbell and Meyer 2006).

The discovery of the transient receptor potential (TRP) family led to a better understanding of how nociceptors detect external stimuli and how they can be sensitized (Caterina et al. 1997). TRP channels are activated by various nociceptive physical and chemical stimuli, providing the generator potential to activate VGSC resulting in ectopic discharge (reviewed in Mickle et al. 2015). Proinflammatory mediators enhance TRPV1 channel function via phosphorylation, provoking peripheral sensitization. Sensitized TRPV1 gets activated by minimally acidic pH and at body temperatures, leading to sustained generator potentials and electrical discharge. Expression of TRPV1 can also be upregulated by nerve damage and the increased inflammatory microenvironment (reviewed in Mickle et al. 2015, 2016). Translocation of TRPV1 to the cell surface also increases the channel activity. Activation of TRPV1 results in membrane depolarization with subsequent AP generation via VGSCs; TTX-insensitive sodium channels can also be sensitized via phosphorylation by protein kinases A and C (Gold et al. 1996).

Neural damage provokes highly organized neuroimmune interactions in peripheral nerves that play a key role in initiating many cellular mechanisms underlying persistent NP (reviewed in Costigan et al. 2009; Marchand et al. 2005; Scholz and Woolf 2007). Accumulation of infiltrating immune cells such as neutrophils, macrophages, and mast cells at the injured site contributes to peripheral sensitization in most neuropathic conditions (Ren and Dubner 2010). They release substances (e.g., NO, ATP, lipids prostaglandins, cytokines, etc.), which sensitize the remaining intact axons and contribute to axonal damage. Schwann cells secrete nerve growth factor (NGF) and matrix metalloproteinases (MMPs) that contribute indirectly to central sensitization (see below). Neuropeptides from nociceptive axons, kinins, and nitric oxide cause a local increase in blood flow and tissue swelling. This neurogenic neuroinflammation affects the micromilieu in the nerve. After the damaged nerves are removed by phagocytosis, neuropathic sensitivity is then maintained by intact axons. Remarkably, similar changes also occur in the dorsal root ganglion (DRG).

### Spinal sensitization

The IASP defines central sensitization as an “*increased responsiveness of nociceptive neurons in the CNS to their normal or subthreshold afferent input*” (Loeser and Treede 2008). The main reason for central sensitization in peripheral NP is the persistent nociceptive afferent input after peripheral nerve damage (Haroutounian et al. 2014). Blocking the afferent input, even in patients with profound signs of central sensitization, temporarily abolishes NP symptoms (Gracely et al. 1992). Patients with NP show different signs of central sensitization, including a pattern of hyperalgesia similar to secondary hyperalgesia (i.e., an increase in pain sensitivity outside the area of injury).

Alterations in calcium permeability, gene expression patterns, phosphorylation of ion channels, neuronal plasticity, and the misbalance between descending facilitation and inhibition promote central sensitization (Latremoliere and Woolf 2009). In animal models of peripheral nerve injury, activation of several protein kinases leads to phosphorylation of ionotropic and metabotropic glutamate receptors and subsequently to enhanced excitatory postsynaptic potential frequency and amplitude (Choi et al. 2017; Hildebrand et al. 2016). Ion channel alterations, such as upregulation of the α2δ-1 subunit of voltage-gated calcium channels (Luo et al. 2001), occur after peripheral nerve damage.

Long-term potentiation (LTP), an activity-dependent persistent synaptic strengthening, intensively studied in the hippocampus, appears to play a role in spinal sensitization after noxious input (Ji et al. 2003; Sandkuhler 2007). There is still no proof of LTP in NP patients, but there are several lines of evidence in favor: conditioning electrical stimulation of the same type that induces LTP in rodents has been shown to induce long-lasting amplification of pain perception in humans (Klein et al. 2004). Brief application of high-dose opioids reversed activity-dependent LTP at C-fiber synapses in preclinical studies (Drdla-Schutting et al. 2012). Further studies need to investigate whether inhibition of LTP can also outlast drug effects in NP patients, which would suggest reversal of LTP and hyperalgesia.

Increased *N*-methyl-d-aspartate receptor (NMDAR) activity contributes to central sensitization after nerve damage. Activation of intracellular pathways by protein kinases leads to phosphorylation of NMDARs. Afterwards, NMDARs respond stronger to agonists. Under normal circumstances, NMDA receptor channels are blocked by Mg^2+^ ions. Phosphorylation by protein kinase C increases the opening probability and decreases the affinity of NMDARs for extracellular Mg^2+^ (Chen and Huang 1992). Activation of protein kinase C also facilitates the upregulation of NMDAR activity and enhances LTP (Lu et al. 1999).

Activation of NMDARs boosts synaptic efficacy and causes Ca^2+^ influx, which can activate intracellular signaling pathways that initiate and maintain central sensitization. Targeting α2δ-1-bound NMDARs with gabapentinoids or α2δ-1 C-terminal peptides can attenuate nociceptive drive from primary sensory nerves to dorsal horn neurons in NP (Chen et al. 2018).

### Involvement of microglia in spinal sensitization

In the last decade, a growing body of literature has delineated neuronal interactions with non-neuronal cells and both their contributions to NP, particularly focusing on neurogenic neuroinflammation (i.e., inflammatory reactions in response to neuronal activity) (Xanthos and Sandkühler 2014). While most studies on diseases of the CNS focus on how microglial-driven neurodegeneration develops, pain researchers turned to investigate mediators released by microglia that modulate synaptic transmission (Salter and Stevens 2017; Woolf and Salter 2000). Since the first role on the specific role of microglia in NP (Jin et al. 2003; Raghavendra et al. 2003; Tsuda et al. 2003), evidence has grown on the role of microglia in preclinical models of NP (Clark and Malcangio 2012; Inoue and Tsuda 2018; McMahon and Malcangio 2009; Tsuda et al. 2005), the contribution of astrocytes is less clear. Since there is now great interest in targeting neuroinflammation to treat NP conditions, some of the neuronal microglial signaling pathways will be presented.

Microglia, the macrophages of the CNS, are found massively in the dorsal horn close to central terminals of damaged afferents (Beggs and Salter 2007) soon after peripheral nerve injury. This activation is caused by several mediators acting on microglial receptors, e.g., ATP acting on P2X4 and P2X7 (Bernier et al. 2018; Inoue 2017; Tsuda et al. 2003) or the two chemokines fractalkine (CX3CL1) and CCL2 acting on their specific receptors (CX3CR1, CCR2) (Clark and Malcangio 2014; Milligan et al. 2008; Thacker et al. 2009; Zhuang et al. 2007). Toll-like receptors are also involved in microglial activation (reviewed in Lacagnina et al. 2018). Subsequently, microglial phenotype changes from a surveillance state to an activated state and several intracellular signaling cascades are activated, e.g., phosphorylation of p38 mitogen-activated protein kinase (MAPK) (Jin et al. 2003). As a consequence, microglia release proinflammatory mediators such as tumor necrosis factor-alpha (TNF-alpha) (Schafers et al. 2003), interleukin 1β (IL-1β) (Gruber-Schoffnegger et al. 2013), and brain-derived neurotrophic factor (BDNF) (Coull et al. 2005) that establish a positive feedback loop during nociceptive signaling and modulate spinal neurons leading to enhanced synaptic transmission (reviewed in Ji et al. 2013; Tsuda et al. 2005). Blocking microglial activation can prevent chronic pain, but cannot reverse it (Raghavendra et al. 2003; Zhang et al. 2017).

In humans, direct evidence of glial activation and its contribution to pain pathogenesis is scarce, but there is evidence of increased levels of proinflammatory mediators in cerebrospinal fluid (e.g., chemokines, TNF-alpha, IL-6) as well as low levels of the anti-inflammatory mediator IL-10 supporting the idea of central neuroinflammation in NP patients (Backonja et al. 2008; Backryd et al. 2017; Kotani et al. 2004; Sun et al. 2017). Elevated levels of a neuroinflammation marker translocator protein (TSPO) with in vivo PET/MR imaging in patients with several chronic pain states including lumbar radiculopathy were demonstrated (Albrecht et al. 2018).

### Supraspinal changes

Hyperexcitability of neurons in nociceptive pathways (Patel and Dickenson 2016) and ion channel alterations (Shen et al. 2015; Wang et al. 2015) can also be found in higher brain regions in NP. Ectopic discharge in the CNS following neuronal disinhibition has been suggested (Keller et al. 2007) and thalamic bursting discharge of patients with central NP may represent such ectopic activity (Lenz et al. 1994). Microglial activation occurs in the thalamus, sensory cortex, and amygdala of the nociceptive pathways after peripheral nerve damage (Taylor et al. 2017). This glial activation leads to enhanced synaptic plasticity in the primary somatosensory cortex, resulting in mechanical hypersensitivity (Kim et al. 2016). Cellular events occurring during glial activation in the periaqueductal gray may also promote descending facilitation during NP (Ni et al. 2016).

Descending pathways from the anterior cingulate gyrus, amygdala, and hypothalamus modulate the spinal transmission via brain stem nuclei in the periaqueductal gray and rostroventral medulla involving neurotransmitters such as norepinephrine, serotonin, and endogenous opioids. Under physiological conditions, there is a balance between descending facilitation and inhibition with a predominance of inhibition. Descending inhibition is at least partly mediated by spinal interneurons that act pre- or postsynaptically at the synaptic transmission from primary afferents to dorsal horn neurons (Zeilhofer et al. 2012). Under pathological conditions, several mechanisms lead to reorganization in these pathways, including an altered transmembrane anion gradient (Keller et al. 2007), microglial-driven downregulation of potassium chloride cotransporters (Coull et al. 2005), loss of GABAergic interneurons (Moore et al. 2002; Scholz et al. 2005), impaired noradrenergic inhibition (Rahman et al. 2008) and increased descending serotoninergic facilitation (Bee and Dickenson 2008).

In human studies, conditioned pain modulation (CPM) gives insight into endogenous descending inhibition and facilitation (Gasparotti et al. 2017; Kennedy et al. 2016; Granovsky 2013). In healthy volunteers, inhibitory effects dominate. Studies comparing healthy volunteers with patients with peripheral polyneuropathy have demonstrated significantly impaired CPM in nondiabetic painful neuropathy (Tuveson et al. 2007) and in pDN patients (Granovsky et al. 2017). CPM can predict the success of pain therapy (Bosma et al. 2018; Yarnitsky et al. 2012) and increasing CPM efficacy can also alleviate pain (Schuh-Hofer et al. 2018).

Neuroimaging studies have shown multiple changes in activity and functional connectivity in CNS regions involved in pain processing and pain modulation (Moisset and Bouhassira 2007). To date, there is no agreement on whether central sensitization acts only as an amplifier of peripheral signals (Meacham et al. 2017) or as an independent pain generator in peripheral NP conditions (Ji et al. 2018). Nevertheless, central mechanisms are essential for the maintenance and chronification of NP (Latremoliere and Woolf 2009).

## Assessment of peripheral neuropathic pain

Neuropathic pain (NP) describes a group of syndromes with many different causes and varying clinical manifestations. Diagnostic algorithms differ depending on whether the underlying lesion or disease is in the peripheral or central nervous system. Hence, a first subdivision of NP is peripheral versus central NP (Scholz et al. 2019). The basic diagnostic approach (i.e., according to the grading system) is the same (Treede et al. 2008; Finnerup et al. 2016), but assessment tools are different (e.g., punch skin biopsy for peripheral vs. MR imaging for central NP).

### Grading system for neuropathic pain assessment

The Neuropathic Pain Special Interest Group (NeuPSIG) of the International Association for the Study of Pain (IASP) issued diagnostic criteria for NP, the Neuropathic Pain Grading System, developed to determine the level of certainty that a patient’s pain is neuropathic in nature or has a neuropathic component in mixed pain syndromes (Finnerup et al. 2016; Treede et al. 2008); it was intended to be used for clinical diagnostics as well as clinical research. This diagnostic approach was also included in the assessment guidelines for NP (Cruccu and Truini 2017; Deng et al. 2016) and in ICD-11 (Scholz et al. 2019). The stepwise approach is based on the history of the patient, physical examination, and confirmatory tests (Table 4). The initial grading system (Treede et al. 2008) struggled with the paradox that classical trigeminal neuralgia is not associated with sensory deficits in the painful area, yet is one of the commonly accepted peripheral NP syndromes. When evoked paroxysms of trigeminal neuralgia had been re-conceptualized as sensory signs (Cruccu et al. 2016), the following hierarchical sequence of four steps could be established in the revised grading system (Finnerup et al. 2016):

**Table 4:** A stepwise approach facilitates the classification of patients’ pain as neuropathic

Diagnostic step	Outcome	Conclusion
*History*		
(1) History of relevant neurological lesion or disease (2) And pain distribution, which is neuroanatomically plausible	Both criteria “yes”	‘Possible neuropathic pain’
*Examination*		
(3) Pain is associated with sensory signs in the same neuroanatomical plausible distribution	Positive results in BSE or QST^a^	‘Probable neuropathic pain’
*Confirmatory tests*		
(4) Diagnostic test confirming a lesion or disease of the somatosensory nervous system explaining the pain^b^	Confirmed	‘Definite neuropathic pain’

Stepwise approach for diagnosis of NP according to the Neuropathic Pain Grading System (Treede et al. 2008; Finnerup et al. 2016)

The levels “probable” and “definite” are both considered to establish the diagnosis, whereas the level “possible” is not

^a^Usually signs of sensory loss, but also allodynia (touch evoked or thermal). *BSE* bedside examination, *QST* quantitative sensory testing

^b^Different for peripheral neuropathic pain (blood glucose levels, HbA1c, nerve conduction studies, surgical evidence, etc.) or central neuropathic pain (MRI, CSF analysis, etc.)

Step 1: The medical history of the patient needs to suggest a lesion or disease that is capable of causing NP. Step 2: Pain distribution is plausible for the underlying lesion or disease (according to, e.g., pain drawing of the patient). When these two conditions are met, the possibility of NP is considered possible (*possible NP*). A detailed clinical examination should then be performed to find confirmatory evidence for the pain distribution and the underlying lesion or disease. Step 3: Since there is no confirmatory test for the spatial extent of perceived ongoing pain, the spatial extent of sensory signs is used as a surrogate. If this condition is also met, the neuropathic nature of the pain is considered to be likely (*probable NP*). Step 4: Depending on the suspected lesion or disease, appropriate confirmatory tests are performed. When positive, they lead to the diagnosis of “*definite NP*”. The level “*probable NP*” is considered sufficient to initiate treatment. The level “*definite NP*” indicates that a physician is able to confirm that the patient has a neurological lesion or disease that might explain his/her pain (Finnerup et al. 2016).

The steps in the grading system follow the usual algorithm of neurological diagnostics and are primarily based on clinical examination. Thus, the experience and skills of the physician who does the assessment are of importance and may be limiting. Most available guidelines agree with this, but applicability and usefulness for the day-to-day clinical setting are limited by test–retest reliability of clinical assessment (Cruccu and Truini 2017; Deng et al. 2016). It should be noted that even the level ‘definite neuropathic pain’ does not mean that causality has been established; it refers to the fact that a physician is able to confirm that the patient has a neurological lesion or disease that might explain his/her pain (Finnerup et al. 2016). Lack of confirmation may, however, lead to underdiagnosing NP in patients with pain as their main or only symptom (Bouhassira and Attal 2011; Cruccu et al. 2016; Finnerup et al. 2016; Scholz et al. 2019). The level “probable NP” is hence considered sufficient to initiate treatment.

### Screening as a first step towards diagnosis

Screening tools for NP are patient-reported questionnaires mostly based on pain descriptors or combined questionnaires and simple clinical tests (Table 5, see also Colloca et al. 2017; Attal et al. 2018). They are widely used in daily clinical practice, especially by non-specialists to initiate necessary further diagnostic assessment (Haanpaa et al. 2011). They are also popular in clinical research due to their simplicity and low cost. Screening tools had different objectives when being developed, and validity is inconsistent, as different reference standards were used (old vs. current definition of NP). The value of a screening tool also depends on reliability, sensitivity for changes, usability in another language after thorough translation, and cross-cultural adaptation process.

**Table 5:** Tools for identification and evaluation of symptoms of neuropathic pain and (painful) diabetic neuropathy

Abbreviation	Full name	Objective(s)	Description	References
*Neuropathic pain*				
DN4	Douleur Neuropathique en 4 Questions	To compare the clinical features of *neuropathic and non-neuropathic pain*	*Clinician-administered* questionnaire (10 items): 7 sensory descriptors and 3 clinical signs related to bedside sensory examination, to be tested by the physician	Bouhassira et al. (2005) Spallone et al. (2012)
DN4-interview	Douleur Neuropathique en 4 Questions-Interview	To compare the clinical features of *neuropathic and non-neuropathic pain*	*Clinician-administered* questionnaire (7 sensory descriptors)	Bouhassira et al. (2005) Spallone et al. (2012)
NPSI	Neuropathic Pain Symptom Inventory	To evaluate the *different dimensions of symptoms of neuropathic pain*	*Patient self-administered* questionnaire (12 items): items related to different pain descriptors (e.g., burning, electric-shock like, squeezing, tingling)	Bouhassira et al. (2004) Crawford et al. (2008) Lucchetta et al. (2011)
PainDETECT	PainDETECT	Screening for the presence of *neuropathic pain* without physical examination	*Patient self-administered* questionnaire (10 items): 1 item time course, 1 item pain intensity, 1 item pain radiation, 7 items pain descriptors (quality)	Freynhagen et al. (2006) Themistocleous et al. (2016)
*Painful diabetic neuropathy*				
DN4	Douleur Neuropathique en 4 Questions	To compare the clinical features of *neuropathic and non-neuropathic pain*	*Clinician-administered* questionnaire (10 items): 7 sensory descriptors and 3 clinical signs related to bedside sensory examination, to be tested by the physician	Bouhassira et al. (2005) Spallone et al. (2012)
DN4-interview	Douleur Neuropathique en 4 Questions-Interview	To compare the clinical features of *neuropathic and non-neuropathic pain*	*Clinician-administered* questionnaire (7 sensory descriptors)	Bouhassira et al. (2005) Spallone et al. (2012)
mBPI-DPN	Modified Brief Pain Inventory	Modified version of the Brief pain Inventory for patients with *painful diabetic neuropathy*	*Patient-completed* numeric rating scale to assess the severity of pain, the impact on daily functioning and other aspects of pain. A modification was made to distinguish between pain attributable to diabetic polyneuropathy and attributable to other causes.	Zelman et al. (2005)
NSC-score	Neuropathy Symptom and Change Score	To detect and grade the severity of *diabetic neuropathy* and *pain*	*Clinician-administered* questions about the type of pain or slight illness, location of symptoms, time of symptom, arousal from sleep and maneuvers that are relieving patients’ symptoms	Xiong et al. (2015)
NTSS-6	Total Symptom Score 6	To evaluate the frequency and intensity of *neuropathic sensory and pain symptoms* in patients with *diabetic peripheral neuropathy*	*Clinician-administered* 6-item questionnaire: frequency and intensity of: numbness and/or hyposensitivity; prickling and/or tingling; burning; aching pain and/or tightness; sharp, shooting, lancinating pain; and allodynia and/or hyperalgesia)	Bastyr et al. (2005)
PainDETECT	PainDETECT	Screening for the presence of *neuropathic pain* without physical examination	*Patient self-administered* questionnaire (10 items): 1 item time course, 1 item pain intensity, 1 item pain radiation, 7 items pain descriptors (quality)	Freynhagen et al. (2006) Themistocleous et al. (2016)
*Diabetic neuropathy*				
CSS	Clinical screening score	To screen T2DM patients for *sensorimotorpolyneuropathy* and need for in-depth foot examination	*Clinician-administered* evaluation of risk factors, diastolic blood pressure, creatinine serum levels, foot inspection and interview for pain and neuropathic symptoms	Bongaerts et al. (2015)
DNE	Diabetic Neuropathy Examination Score	To diagnose *distal diabetic polyneuropathy*	*Clinician-administered* (8 item) examination about muscle strength, reflexes and sensations (pinprick, SWMF, vibration and proprioception)	Meijer et al. (2000) Meijer et al. (2003) Liyanage et al. (2012)
DNS	Diabetic Neuropathy Symptom Score	To assess *distal neuropathy* in patients with *diabetes*	*Clinician-administered* (4 item) symptom score: 1. Unsteadiness in walking, 2. Pain, burning or aching at legs or feet, 3. Prickling sensations in legs or feet and 4. Numbness in legs or feet	Meijer et al. (2002) Liyanage et al. (2012)
mTCNS	Modified Toronto Clinical Neuropathy Score	To modify the TCSS to better capture a categorical scale of simple sensory tests which are representative of the early dysfunction in *diabetic sensorimotorpolyneuropathy*	*Clinician-administered* symptom scores and sensory test scores	Bril et al. (2009)
MNSI	Michigan Neuropathy Screening Instrument	To screen large numbers of patients in a routine clinical setting for the presence of *diabetic neuropathy* Patients who screen positive on the MNSI may be referred for the administration of the MDNS	Section A: *self‐administered by the patient* through 15 “yes” or “no” questions about foot sensation, numbness, temperature alterations, general asthenia, and peripheral vascular disease Section B: based on clinical examination (*clinician*-*administered*): (1) inspection of both feet (2) examination and grading of muscle stretch reflexes (3) determination of vibration sensation	Feldman et al. (1994) Rahman et al. (2003) Moghtaderi et al. (2006) Xiong et al. (2015) Barbosa et al. (2017) Sartor et al. (2018)
MDNS	Michigan Diabetic Neuropathy Score	To provide a means of diagnosing and staging *diabetic neuropathy* that is simpler and less time consuming than accepted research protocols	*Clinician-administered* sensory impairment testing, muscle strength testing and reflexes	Feldman et al. (1994)
NDS	Neuropathy Disability Score	To detect *deficits affecting the peripheral nervous system*	*Clinician-administered* evaluation of cranial nerves, muscle weakness, reflexes and loss of sensations	Dyck et al. (1980)
Norfolk QoL-DN	Norfolk Quality of life questionnaire – diabetic neuropathy	To capture the entire spectrum of *diabetic neuropathy* including sensory loss of function, balance, motor impairments and autonomic symptoms	*Patient self-administered* description of symptoms and complications and their duration, generic health status	Vinik et al. (2005)
NSS	Neuropathy Symptom Score	To detect and grade the severity of *diabetic neuropathy* based on a recorded evaluation of neurological symptoms	*Clinician-administered* testing of muscle weakness, sensory disturbances, and autonomic signs	Dyck (1988) Dyck et al. (1980)
TNS	Total Neuropathy Score	To grade severity of *diabetic polyneuropathy*	*Clinician-administered* composite measure of peripheral nerve function combining the grading of symptoms, signs, nerve conduction studies and quantitative sensory testing	Cornblath et al. (1999)
TCSS	Toronto Clinical Scoring System	To examine the presence and severity of *diabetic peripheral sensorimotor polyneuropathy* as assessed via electrophysiological criteria and myelinated fiber density on sural nerve biopsy	*Clinician-administered* classical neurological history (symptom scores) and examination techniques (reflex scores and sensory test scores) and designed to be simple and relevant to the clinician	Bril and Perkins (2002)

Validity is inconsistent and not fully convincing, as different reference standards were used. Thus, validity is not always sufficient for daily clinical practice. In these tests, pDN is often not included in validation, mostly only neuropathic symptoms are assessed but not pain in particular. This table is not exhaustive. References refer to first description of the instrument and/or, if available, to validation studies in diabetic patients

The DN4 has been validated in a population of patients with painful diabetic neuropathy (pDN) (Spallone et al. 2012), which was defined as “*the presence of diabetic polyneuropathy plus chronic neuropathic pain in the same area as neuropathic deficits*”; NP was assessed based on pain history and examination, which is consistent with the grading system. DN4 showed a sensitivity of 80% and a specificity of 92%. Another study compared the DN4 and the PainDETECT with the NeuPSIG definition and grading system as the reference standard; it resulted in a sensitivity and specificity for the DN4 of 88% and 93% and for the PainDETECT of only 61% and 92% (Themistocleous et al. 2016).

In a recently published systematic review regarding measurement properties of different screening tools for NP it was concluded that the Neuropathic Pain Questionnaire (NPQ) (Krause and Backonja 2003) and the DN4 (Bouhassira et al. 2005) were the most suitable for use in daily clinical practice (Mathieson et al. 2015). However, screening tools developed before 2008 (e.g., PainDETECT; Freynhagen et al. 2006) were validated against an obsolete definition of NP (“dysfunction” instead of “lesion or disease”), but not against the current definition of NP as endorsed by NeuPSIG (Treede et al. 2008), IASP (Jensen et al. 2011) and WHO (Scholz et al. 2019). DN4 and PainDETECT correlate only moderately against the grading system (Timmerman et al. 2017, 2018a; Epping et al. 2017; Tampin et al. 2013). This might lead to inconclusive results in prevalence studies and inaccurate clinical diagnostics and hence, improper treatment. Therefore, screening cannot replace thorough physical examination (Timmerman et al. 2017).

### Bedside examination for diabetic neuropathy and neuropathic pain

Bedside examination (BSE) in patients with DM is essential when suspecting diabetic polyneuropathy (dPNP) and/or pDN. Most guidelines advise yearly screening for dPNP (in T1DM starting 5 years after diagnosis, in T2DM starting immediately after diagnosis; Pop-Busui et al. 2017; German National Disease Management Guideline for Diabetic Neuropathy). A thorough clinical examination, including inspection of the feet, evaluation of sensory loss, arterial pulses, skin state, pain assessment, and BSE as described below is an advisable basis. For the vast majority of patients, the diagnosis of dPNP is based on history and examination, without further necessary testing.

A typical BSE test in patients suspected for dPNP is the 128 Hz tuning fork (placed at the dorsum of the interphalangeal joint of the hallux) to examine vibration perception. It is a valid and reliable tool for screening purposes, manageable in daily clinical practice (Meijer et al. 2005). Additionally, testing by monofilaments is easily applicable and has a reliable outcome. Two studies (Olaleye et al. 2001; Perkins et al. 2001) found the following BSE tests useful to differentiate between DM patients with and without neuropathy: The Semmes–Weinstein 10 g monofilament examination (SWME), the superficial pain sensation (via a sterile neurotip) and vibration (on–off method). Nerve Conduction Studies (NCS), often a reference standard versus screening instruments, were also suggested to be included in annual screening for dPNP (Perkins et al. 2001). However, there is some evidence that one test alone is not sufficient (Brown et al. 2017) and that NCS may be replaced by QST profiling (Kopf et al. 2018).

BSE for pDN and NP, in general, should include a pain drawing by the patient (Hansson 2002; Margolis et al. 1986) and mapping of regions of sensory disturbances using at least one thermal and one mechanical test stimulus (Timmerman et al. 2018b; La Cesa et al. 2015; Haanpaa et al. 2011; Bouhassira and Attal 2011; Cruccu et al. 2010; Haanpaa et al. 2009). According to the grading system, sensory changes should be documented within the painful region for grading of “probable NP”. For a review including a well-designed table giving an overview of negative and positive symptoms of NP, see Gierthmuhlen and Baron (2016).

### Confirmatory tests

There are two types of confirmatory tests in the assessment of patients with NP: (a) tests that confirm the sensory changes and (b) tests that confirm the specific underlying lesion or disease of the somatosensory nervous system explaining the symptoms of the patient (Brown et al. 2017; Finnerup et al. 2016; Olaleye et al. 2001; Perkins et al. 2001).

A number of confirmatory tests to investigate somatosensory pathway function are available (Table 6 including a column with remarks on the application in dPNP). They can be divided into structural tests (nerve biopsy, punch skin biopsy, corneal confocal microscopy) and functional tests (quantitative sensory testing, neurophysiological techniques). These tests are used mostly in research settings or in the diagnostic workup of patients with an atypical clinical presentation (Feldman et al. 2019; Tesfaye et al. 2010).

**Table 6:** Confirmatory tests for lesion or disease of somatosensory system in patients with suspected neuropathic pain

Name	Objective(s) of test	Description	Remarks on dPNP	References
Basic neurological examination	Mapping of sensory changes	Inspection of feet, evaluation of clinical signs (e.g., sensory loss, allodynia, hyperalgesia), pulse state, skin state, general state of patient, reflexes etc	Recommended in all guidelines. Essential for grading of NP in all patients.	Holiner et al. (2013) German National Disease Management Guideline for Diabetic Neuropathy Pop-Busui et al. (2017) Cruccu et al. (2010)
Quantitative sensory testing (QST)	Quantification of sensory changes in a few defined areas	Mechanical and thermal detection and pain thresholds to assess small (C and Aδ) and large (Aβ) sensory nerve fibers	QST is proven to be reliable and reproducible, and sensitive to change in NP, also in diabetic patients.	Treede (2019) Rolke et al. (2006) Cheliout-Heraut et al. (2005) Weintrob et al. (2007) Hsieh (2010) Backonja et al. (2013) Jensen et al. (1991)
NerveCheck	A portable QST device	Vibration and thermal testing for functional testing of large and small nerve fibers	Validated against neuropathy disability score, nerve conduction studies, intraepidermal and corneal nerve fiber density.	Ponirakis et al. (2016)
Ankle reflexes	Assess muscle spindle afferents and Aα motoneurons	Tendon tap by reflex hammer; assesses only large fiber functions	Loss of ankle reflexes occurs early in dPNP. Part of recommended clinical examination.	Tesfaye et al. (2010) Pop-Busui et al. (2017)
Nerve conduction studies (NCS)	Estimating severity of diabetic neuropathy by testing motor (Aα) and large sensory (Aβ) nerve fibers	Usually NCs of sural nerve; objective and quantitative measure	Changes in amplitude of motor nerve fibers typically follow changes in amplitude of sensory nerve fibers. If NCS is normal, validated measures of small fiber neuropathy are needed.	Tesfaye et al. (2010) Dyck et al. (1993) Dyck et al. (2010) Dyck et al. (2011) Apfel et al. (2001)
Laser-evoked potentials (LEPs)	Testing small fiber function (Aδ and C): thermo-nociceptors	Laser heat pulses on hairy skin; easiest and most reliable technique for objective assessment of nociceptive fibers	Validated for detection of small fiber neuropathy against skin punch biopsy. Diagnostic accuracy in diabetic small fiber neuropathy is established.	Di Stefano et al. 2017) Cruccu et al. 2008)
Cold evoked potentials	Small fiber function: thermoreceptors	Objective test for thermoreception by contact stimulator	Note: validity and role in routine diagnostic are not yet established!	De Keyser et al. (2018) Leone et al. (2019) Farooqi et al. (2016)
Axon reflex flare response	Efferent function of small nociceptive nerve fibers	Stimulation of peptidergic C-fibers by iontophoresis or heat, assessment of vasodilation by laser Doppler imaging	Reduced in subjects with impaired glucose tolerance and type 2 diabetic patients with and without neuropathy.	Caselli et al. (2003) Krishnan and Rayman (2004)
Neuropad	Evaluate cholinergic small sympathetic nerve fiber function	A simple visual indicator test based on sweating and on color change	Test for autonomic neuropathy.	Ponirakis et al. (2014)
Intraepidermal nerve fiber density (IENF)	Gold standard for the structural diagnosis of small­fiber neuropathy (skin punch biopsy)	Acquired by skin punch biopsy or blister technique at ankle	Invasive, rarely used in routine diagnostic, only advised when NCS and QST are normal. IENF density correlates inversely with both cold and heat detection thresholds.	Vlcková-Moravcová et al. (2008) Nebuchennykh et al. (2009) Shun et al. (2004) Andersson et al. (2016) Devigili et al. (2008) Sorensen et al. (2006) Alam et al. (2017)
Corneal confocal microscopy	Structural diagnosis of small fiber neuropathy	Autofluorescence of corneal nerve fibers; corneal anesthesia required	Correlates with IENF loss and the severity of dPNP, was more prominent in patients with pDN.	Quattrini et al. (2007) Mehra et al. (2007) Alam et al. (2017)
Nerve biopsy	Structural diagnosis of large fiber neuropathies	Usually biopsy of the sural nerve	Invasive and highly specialized procedure requiring electron-microscopy. Not recommended for routine use.	Malik et al. 2005) Quattrini et al. (2007)

Confirmatory tests have different scopes (sensory loss, surrogate nerve damage, morphology of peripheral nerve endings). Pain is per definition a subjective experience (IASP) and thus does not have a confirmatory test itself (Davis et al. 2017). This table is not exhaustive

For all confirmatory tests, reference values have to be adjusted for test site, age, sex, and population. For quantitative sensory testing (QST), multi-center reference data are available for different body regions in both sexes and a broad age range (Magerl et al. 2010; Pfau et al. 2014; Vollert et al. 2016). These reference data allow a transformation of a patient’s data into Z-scores with a standard Gaussian distribution (zero mean and unity variance), provided the examiner has calibrated herself or himself for about 20 healthy subjects (Vollert et al. 2016). There are also some reference data available for non-Caucasian populations (Gonzalez-Duarte et al. 2016; Ezenwa et al. 2016). For NCS, each laboratory is usually required to generate its own reference data. Published reference data can only be used for a broad orientation since the techniques are not standardized well enough, e.g., a normative database from a mainly Western population (USA) was found not to be suitable for a Japanese population (Hirayasu et al. 2018).

Importantly, note that also “objective” instruments such as skin biopsies do not necessarily relate to the pain complaint of the patient. Although assessment of intraepidermal nerve fiber density (IENFD) through skin biopsy is validated for diagnostics of small fiber neuropathies, including dPNP (Lauria et al. 2010; Tesfaye et al. 2010), the correlation between IENFD and severity of NP in dPNP is still debated. A flooring effect of IENFD has been suggested (Cheng et al. 2013; Shun et al. 2004; Sorensen et al. 2006; Themistocleous et al. 2016; Truini et al. 2014).

Finally, not every neuropathy in patients suffering from DM is a diabetic neuropathy. Dependent on clinical presentation, laboratory testing is advised to exclude differential diagnosis, such as thyroid disease, autoimmune disorders, infections (e.g., HIV), vitamin deficiencies (e.g., vitamin B12) or intoxications (e.g., alcohol). Thus, diabetic neuropathy is a diagnosis of exclusion (England et al. 2005; Pop-Busi et al. 2017; Ziegler et al. 2014).

## Pathophysiology of diabetic neuropathy

### General aspects

Diabetes mellitus (DM) is a common disorder marked by persistent hyperglycemia and other metabolic disturbances, such as dyslipidemia. Increased glucose levels affect primarily cells that have a limited capacity to regulate their glucose intake, including vascular cells, Schwann cells, and neurons of the peripheral and central nervous systems. Consequently, hyperglycemia leads to largely intractable complications such as retinopathy, nephropathy, hypertension, and neuropathy. However, not all patients with DM develop diabetic polyneuropathy (dPNP). In prospective studies with diabetic patients, environmental risk factors for the development of neuropathy in type 1 (T1DM) (Tesfaye et al. 2005) and type 2 diabetes (T2DM) (Andersen et al. 2018) were investigated: They include poor glycemic control (for T2DM Pop-Busui et al. 2013) and common cardiovascular risk factors (e.g., hypertension, raised triglycerides, obesity, smoking). Genetic risk factors might also contribute to the pathogenesis of dPNP (reviewed in Prabodha et al. 2018).

Peripheral diabetic neuropathy can present as several different patterns (see “Clinical presentation of painful diabetic neuropathy”). Damage primarily occurs in sensory neurons, resulting in positive symptoms (e.g., pain, paresthesias) and negative symptoms, such as sensory loss (e.g., numbness). The most common pattern is the distal symmetric polyneuropathy, which clinically presents as a neuropathy not only predominantly of the feet, but also of the hands, with a distal-to-proximal gradient of severity (Callaghan et al. 2012a).

### Pathogenesis of diabetic polyneuropathy

**Fig. 2:**
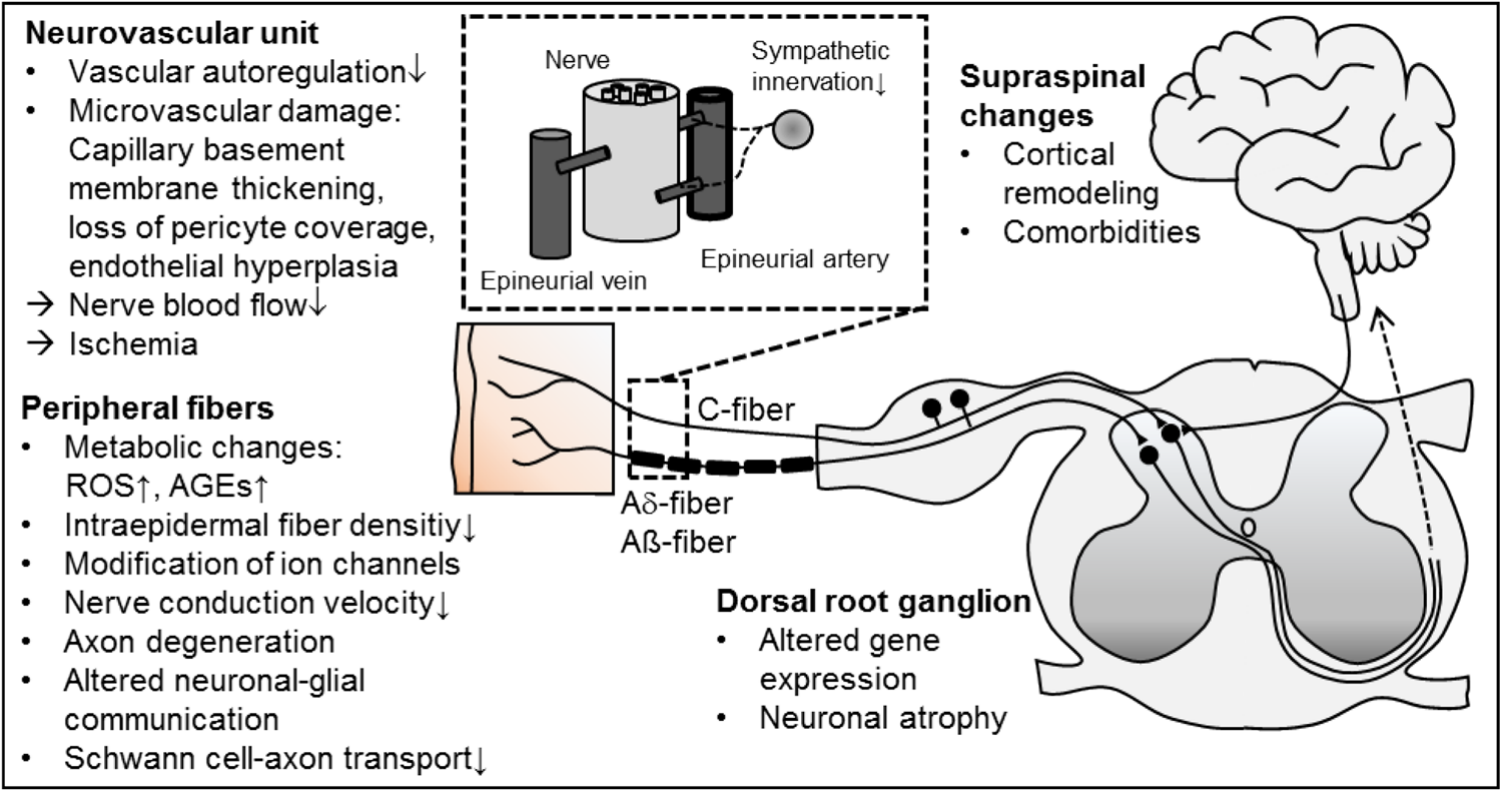
Selection of structural and functional alterations in diabetic neuropathy. *AGE* advanced glycation end products, *ROS* reactive oxygen species

Evidence of the pathogenesis of dPNP has accumulated over the past decades, delineating alterations in neurons, glia, immune cells and vascular cells in the progress of DM leading to loss of peripheral nerve function (Fig. 2; reviewed in Feldman et al. 2019; Feldman et al. 2017; Schreiber et al. 2015; Sloan et al. 2018; Yagihashi et al. 2011). Many metabolic and vascular maladaptive responses have been described. Recently, the research focus turned to uncover interactions with Schwann cells and other non-neuronal cells, endoplasmatic reticulum stress, neurodegeneration, and mitochondrial dysfunction.

Most data were obtained in preclinical studies investigating animal models of T1DM (streptozotocin) and less so of T2DM using diet or genetically induced DM (reviewed in O'Brien et al. 2014; Sullivan et al. 2008). These animals develop primarily distal axon loss, systemic injury of the peripheral nervous system and altered interactions with Schwann cells, which is recognized as a model for dPNP.

Evidence supports that the entire primary sensory neuron is targeted by diabetes. However, it remains elusive whether damage first targets peripheral axons and their associated Schwann cells or the neuron perikarya that reside in the dorsal root ganglia (DRG) where they are not protected by a blood–nerve barrier. Although dPNP is not considered primarily a demyelinating neuropathy, Schwann cells are targeted by chronic hyperglycemia and more severe cases of dPNP in patients include features of demyelination (Feldman et al. 2017; Kobayashi and Zochodne 2018).

Hyperglycemia and dyslipidemia are key players in the development of dPNP. This substrate overload leads to the accumulation of toxic metabolites and mitochondrial dysfunction, promoting metabolic, and oxidative stress and axonal degeneration (Fernyhough 2015; Fernyhough and McGavock 2014). Glucose excess leads to polyol and hexosamine pathway hyperactivity, resulting in both increased reactive oxygen species (ROS) and inflammation, adding to mitochondrial injury (Feldman et al. 2017). Moreover, glycation of numerous structural and functional proteins leads to the production of advanced glycation end-products (AGEs). AGEs result in altered or loss of protein function and interact with AGE-specific receptor modifying gene expression, intracellular signaling, and promoting the release of pro-inflammatory molecules and free radicals (Singh et al. 2014). More recently, researchers have focused on dyslipidemia in mediating additional inflammation and ROS accumulation with continued and progressive nerve injury.

Microvascular alterations induce impaired nerve perfusion provoking hypoxia and loss of nerve function (Tesfaye et al. 1992). Increases in endoneurial capillary density are present in diabetic patients, suggesting that capillary density may respond to diabetes-induced nerve ischemia (Thrainsdottir et al. 2003). Furthermore, altered bioavailability of different mediators including insulin growth factors, vascular endothelial growth factor (Schratzberger et al. 2001) as well as gasotransmitters (e.g., NO, CO, H_2_S) (van den Born et al. 2016) contributes to both vascular disease and neuropathy in diabetes.

Although neurotrophic effects of insulin on sensory nerves have been shown (Frazier et al. 1972), correcting hyperglycemia with insulin has little effect on dPNP in patients with T2DM. By contrast, in T1DM patients with dPNP, normoglycemia through insulin treatment provides a substantial therapeutic benefit (Grote and Wright 2016). Inflammatory processes may be relevant, especially in the onset of dPNP and in developing pDN (Herder et al. 2017; Magrinelli et al. 2015).

### Painful vs. painless diabetic neuropathy

**Fig. 3:**
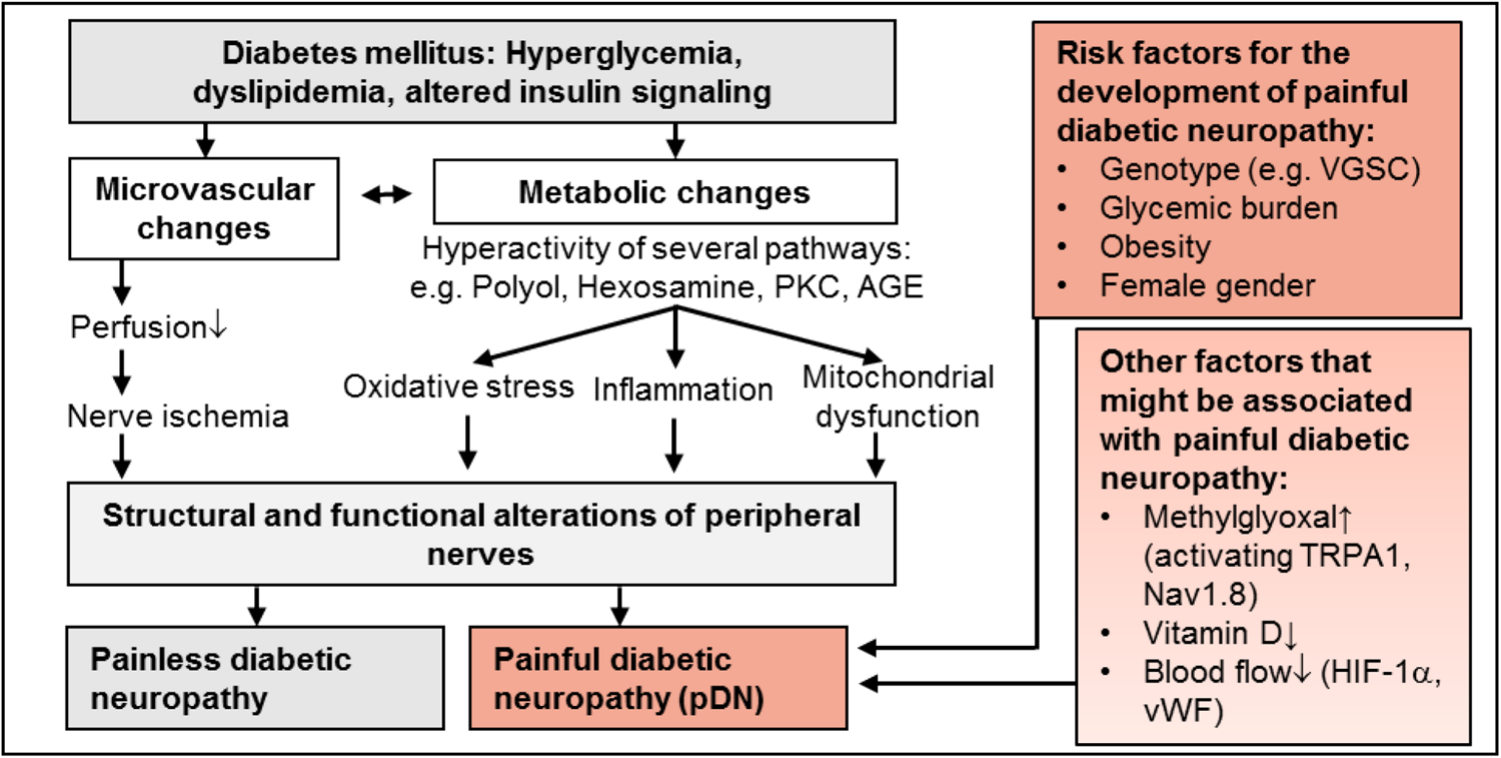
Pathophysiology of painless and painful diabetic neuropathy: diabetes mellitus leads to several pathological changes in neuronal, immune and vascular cells that can lead to structural and functional alterations of the nervous system that can result in diabetic neuropathy (see Fig. 2). Several factors contribute to the development of neuropathic pain in diabetic neuropathy. *AGE* advanced glycation end products, *HIF-1α* hypoxia-induced factor 1α, *PKC* protein kinase C, *TRPA1* transient receptor potential ankyrin 1, *VGSC* voltage-gated sodium channel, *vWF* von Willebrand factor

Only about 20–50% of patients with DM and about 60% of dPNP patients develop NP. The reason why some patients experience NP while others do not is not fully understood. However, there has been growing evidence on risk factors (demographic, metabolic, sensory, genetic) for developing NP in dPNP (Shillo et al. 2019). Large, well-characterized, cross-sectional cohort studies have given valid insights into risk factors (Fig. 3) and somatosensory profiles of pDN. However, longitudinal studies are lacking.

NP in dPNP seems to be associated with the female gender (Truini et al. 2018), increasing age (Van Acker et al. 2009), and ethnicity (Hebert et al. 2017). Metabolic issues including obesity (Ziegler et al. 2018), elevated HbA1c (Themistocleous et al. 2016), high alcohol intake, duration, and type of DM, might increase the risk of developing NP. The sensory phenotype and neuropathy severity are also associated with pDN (Raputova et al. 2017). Genetic variants (Blesneac et al. 2018; Prabodha et al. 2018) and genetic variability are likely to interact with the environment to determine the risk of developing pDN.

#### Peripheral mechanisms of painful diabetic neuropathy

For long time, it has been known that microvascular alterations, such as structural and functional abnormalities of the vasa nervorum (Cameron et al. 2001) and altered regulation of peripheral blood flow (Archer et al. 1984) are associated with pDN. More recently, dysregulation of the local blood flow in the skin, involving hypoxia-induced factor 1-alpha (Quattrini et al. 2008) and von Willebrand factor (vWF) (Shillo et al. 2017), has been found to contribute to NP. NP-related behavior has been found to be related to numerous metabolic pathways. There is limited evidence to support glycemic control or lifestyle modifications in reducing NP (Pop-Busui et al. 2017). Preclinical studies indicate that vitamin D plays a critical role in nerve function in health and possibly in NP syndromes (Fukuoka et al. 2001). Vitamin D levels have been found to be significantly lower in patients with painful compared to painless diabetic neuropathy (Shillo et al. 2019).

There might be a special role for methylglyoxal (MGO) in pDN. In rodent models of pDN, MGO induced signs of hypersensitivity via activation of the sodium channel Nav1.8 and transient receptor potential channel ankyrin 1 (TRPA1) (Bierhaus et al. 2012; Huang et al. 2016). MGO induces pain, axon-reflex-erythema, and long-lasting hyperalgesia via the activation of C-nociceptors in healthy humans. TRPA1 is crucially involved in MGO-induced pain sensation and heat hyperalgesia (Dull et al. 2019).

#### Central mechanisms of painful diabetic neuropathy

Studies of the CNS demonstrate clear differences in painful compared to painless diabetic neuropathy. Spinal, somatomotor, limbic, thalamic, ascending, and descending modulatory systems demonstrate structural and functional alterations (Tesfaye et al. 2016). Changes in higher brain centers are also described in patients with pDN: cortical atrophy within the somatomotor cortex and insula (Selvarajah et al. 2018b; Shillo et al. 2016), abnormal cortical interactions within the somatomotor network (Selvarajah et al. 2018a), and increased cerebral blood flow in the anterior cingulate cortex (Watanabe et al. 2018). It is still unknown whether the described CNS changes are only a response to afferent input of the peripheral nervous system or a primary mechanism responsible for the maintenance of pDN.

## Clinical presentation of painful diabetic neuropathy

Painful diabetic neuropathy (pDN) is defined as “*pain arising as a direct consequence of abnormalities in the peripheral somatosensory system in people with diabetes*” (Tesfaye et al. 2010)*.* According to this guideline, pDN is diagnosed if pain lasted ≥ 3 months has a history of confirmed dPNP and an association with abnormal sensory signs of small fiber and large fiber neuropathy in a neuro-anatomically plausible distal and symmetrical distribution. The stepwise procedure (Table 4) consisting of a history of disease, examination, and confirmatory testing should also be pursued in patients with a suspected pDN.

Distribution of both pain and sensory changes should be mapped (Cruccu et al. 2010; Hansson 2002; Margolis et al. 1986). The sensory changes are usually in a distal, symmetrical distribution (stocking or glove like): decreased sense of vibration, impairment of proprioception, reduced or even absent reflex activity in the Achilles tendon and diminished muscle strength or atrophy of the foot muscles, which might lead to pes cavus or hammertoes (Dyck et al. 1992; England et al. 2005; Martin 1953; Peltier et al. 2014). However, in pDN, symmetrical, distal pain or numbness may also be the only indicative factors (Tesfaye et al. 2010).

Paradoxically, lesions or diseases affecting the somatosensory nervous system do not only lead to a loss of function, but also to overexcitability and increased sensitivity to painful stimuli (hyperalgesia), pain sensations to normally non-painful stimuli (allodynia) and spontaneous pain. pDN patients often describe a prickling, burning, deep aching, sharp, stabbing, and/or electric pain. Signs of sensory gain were traditionally thought to be rare in pDN, but recent studies using QST demonstrated a high prevalence of mechanical hyperalgesia in pDN (Themistocleus et al. 2016; Vollert et al. 2018; Kopf et al. 2018). Preserved small fiber function combined with thermal hyperalgesia (“*irritable nociceptor*” phenotype) is much less frequent in pDN (< 10%) than in postherpetic neuralgia, where it was originally described (Fields et al. 1998; Baron et al. 2017; Edwards et al. 2016; von Hehn et al. 2012). In most pDN patients, sensory loss of small and large fibers is pronounced (“*deafferentiation*” phenotype), while deep tissue mechanical pain (assessed by pressure pain threshold) is relatively intact (Themistocleous et al. 2016; Baron et al. 2017; Vollert et al. 2018).

Small fiber neuropathy in DM may also lead to autonomic nervous system deficits in addition to cutaneous sensory loss and pain. Clinicians need to keep an eye on the usually slower developing autonomic diabetic neuropathy, which affects the cardiovascular, gastrointestinal or genitourinary system (Edwards et al. 2008) and is important for prognosis. In some cases, diabetic neuropathy with neuropathic symptoms may manifest even before DM has been diagnosed (Tesfaye et al. 2010), making diagnostics difficult.

## Implications for management

### Management of diabetic neuropathy

Currently, there are three main elements in the management of diabetic polyneuropathy (dPNP): glycemic control, foot care, and symptomatic treatment, and predominantly pain therapy.

#### Glycemic control

While diabetes is defined by increased glucose levels and tight glycemic control has a high value in its treatment, it is currently recommended to individualize glycemic control in patients with T2DM considering the benefit/risk ratio (Rodriguez-Gutierrez et al. 2016; Qaseem et al. 2018). Hyperglycemia is not the prime driving cause of all complications (Tesfaye et al. 2010). Otherwise, diabetic complications including dPNP could be easily prevented and symptoms should be reduced by efficient glycemic control (Stolar 2010). While there is convincing evidence that enhanced glucose control significantly reduces or delays the incidence of developing clinical neuropathy in T1DM (Fullerton et al. 2014; Albers et al. 2010), the data in the more frequent T2DM remain elusive (Pantalone et al. 2018; Callaghan et al. 2012b; Ismail-Beigi et al. 2010; Calles-Escandon et al. 2010; UKPSD Study Group 1998).

Comparing the effect of glycemic control, Callaghan and colleagues proposed that mechanisms of dPNP in T1DM and T2DM are fundamentally different, which should be considered in its treatment (Callaghan et al. 2012b), still, a recent cohort study emphasizes that early, intensive glycemic control may be necessary to avoid diabetic complications and mortality (Laiteerapong et al. 2019). Interestingly, aggressive glucose control not only significantly increases the risk of severe hypoglycemic episodes (Callaghan et al. 2012b), but can also result in treatment-induced neuropathy if glycemic control is achieved too quickly and to too low glucose levels (Gibbons 2017a, b; Hwang and Davies 2016). It is suggested that neurons take up much more glucose than other cell types and, therefore, are more vulnerable to hypoglycemia (Low and Singer 2015; Gibbons and Freeman 2015).

#### Foot care

dPNP is the primary risk factor for the development of foot ulcers, increasing devastating outcomes of ulcerations that risk amputations (Ahmad 2016; Boulton 2008). Management includes patient education, plantar pressure relief with orthoses and appropriate footwear, regular skin, nail and ulcer care (e.g., paring of calluses, debridement of infected or nonviable tissue) (Pinzur et al. 2005; Kavitha et al. 2014).

### Management of neuropathic pain and painful diabetic neuropathy

Management of neuropathic pain (NP) in general and painful diabetic neuropathy (pDN) in particular is a challenge. A number of clinical practice guidelines, e.g., by the International Association for the Study of Pain (IASP) (Finnerup et al. 2015; Dworkin et al. 2013; Haanpaa et al. 2011), the European Federation of Neurological Societies (EFNS) (Attal et al. 2010; Cruccu et al. 2010), the National Institute for Health and Care Excellence of the UK (NICE; Tan et al. 2010), the Canadian Pain Society (CPS; Moulin et al. 2014), the German Society for Neurology (DGN) and German National Disease Management Guideline for Diabetic Neuropathy have been published to facilitate assessment and management of NP also in patients with DM. Management rests on three pillars: management of diabetes, management of diabetic neuropathy, and symptomatic treatment of NP.

Between these guidelines, there is broad agreement regarding pharmacological management of NP (Finnerup et al. 2015; Deng et al. 2016; Cruccu and Truini 2017).

1. All guidelines strongly recommend three drug classes for first-line therapy of nearly all described syndromes: tricyclic antidepressants (particularly amitriptyline), serotonin–norepinephrine reuptake inhibitors (SNRIs; e.g., duloxetine) and calcium channel alpha-2-delta ligands gabapentin and pregabalin.
2. Second-line recommendations by most guidelines (NICE: only rescue therapy because of higher withdrawal due to adverse events and weak study evidence) is tramadol (weak opioid + SNRI).
3. Third- and fourth-line treatments commonly include strong opioids, anti-convulsants (other than gabapentinoids), and cannabinoids.

Depending on the syndrome, there are some specific recommendations, such as carbamazepine for trigeminal neuralgia or topical agents such as dermal patches releasing lidocaine or capsaicin and subcutaneous injection of botulinum toxin for localized NP (Mick et al. 2011; Allegri et al. 2016). HIV-related NP is more refractory to pharmacotherapy than other types of NP (Finnerup et al. 2015). Opioids should be reserved for patients not responding to therapeutic alternatives because their long-term use carries a high risk of adverse effects and they are effective in the long run only in a small number of patients (for those patients, they may be a valuable element of their multimodal pain management). Nonsteroidal anti-inflammatory drugs have no proven efficacy against pain of purely neuropathic origin; however, they can be useful in mixed pain syndromes (Vo et al. 2009).

The latest NeuPSIG guidelines (IASP) are based on a large meta-analysis, including unpublished trials (Attal and Bouhassira 2015; Finnerup et al. 2015; Cruccu and Truini 2017). These guidelines do not focus on etiologies, but treat NP as a specific entity because the efficacy of systemic treatments seems not to be affected by etiology. This unifying concept has been adopted by EMA for licensing of medications in Europe and is represented in Table 7. The FDA, however, still requests two independent studies per indication for licensing in the U.S.A. Depending on the country, some drugs have not been tested or approved for NP of certain underlying diseases; this needs to be verified locally before following any of these guidelines (Finnerup et al. 2015; Attal et al. 2010; Cruccu et al. 2010; Haanpaa et al. 2011; Dworkin 2010).

**Table 7:** Pharmacotherapy for neuropathic pain

Drug class	Mechanism of action	Representatives	License^a^	Strength of recommendation^b^	NNT^c^	Additional benefits^d^	References
*Anticonvulsants*							
	Calcium channel alpha-2-delta ligand, reduces the synaptic release of several neurotransmitters, channel modulator	Gabapentin	*General NP*	Strong; 1st line	*6.3 (5.0–8.3)* 3.3–7.2	No clinically significant drug interactions additionally for pregabalin: improvement of anxiety and sleep	Goodman and Brett (2017) Dworkin et al. (2010) Moore et al. (2014) Wiffen et al. (2017)
		ER/enacarbil	*General NP*	Strong; 1st line	*8.3 (6.2–13.0)*		
		Pregabalin	*General NP* FDA: pDN	Strong; 1st line	*7.7 (6.5–9.4)* 3.3–8.3		Freynhagen et al. (2005) Roth et al. (2010) Goodman and Brett (2017) Derry et al. (2019)
	Sodium channel	Carbamazepine	*TN*	Inconclusive	Not reported		Collins et al. (2000)
	blocker	Oxcarbazepine					Zhou et al. (2017)
*Antidepressants*							
TCA	Norepinephrine and serotonin reuptake inhibitor, block of voltage-gated sodium channels, anticholinergic effects	Amitriptyline Nortryptilin Desipramine Imipramine	*General NP*	Strong; 1st line	*3.6 (3.0–4.4)* 2.1–4.2	Improvement of depression and sleep disturbances	Saarto et al. (2007) Attal et al. (2010) Dworkin et al. (2010) Finnerup et al. (2010) Moore et al. (2015) Hearn et al. (2014a) Hearn et al. (2014b)
SNRI	Norepinephrine and serotonin reuptake inhibitor	Venlafaxine		Strong; 1st line	*6.4 (5.2–8.4)* 5.2–8.4	Improvement of depression and general anxiety (for both listed drugs)	Zin et al. (2008) Trouvin et al. (2017) Gallagher et al. (2015) Aiyer et al. (2017)
	Norepinephrine and serotonin reuptake inhibitor	Duloxetine	*pDN* FDA: pDN	Strong; 1st line	*6.4 (5.2–8.4)* 3.8–11.0		Snyder et al. (2016) Lunn et al. (2014) Attal et al. (2010) Dworkin et al. (2010) Finnerup et al. (2010) Saarto et al. (2007)
*Local therapeutics*							
Local anesthetics	Sodium channel blocker	Lidocaine (patch, 5%)	*Peripheral NP*	Weak; 2nd line (1st line option in elderly and frail patients)	Not reported	No systemic side-effects	Wolff et al. (2010) Zur et al. (2014) Peltier et al. (2014) Yang et al. (2019)
Local capsaicin	TRPV1 agonist reversible defunctionalization of nociceptor fibers	Capsaicin (patch, 8%)	*Peripheral NP*	Weak; 2nd line	*10.6 (7.4–18.8)*	No systemic side effects	Yang et al. (2019) Derry et al. (2017) Vinik et al. (2016)
Botulinum toxin type A	Acetylcholine release inhibitor, neuromuscular blocking, potentially also mechano-transduction and central effects of NP	BOTOX		Weak, 3rd line	*1.9 (1.5–2.4)*	Potential effect on neurogenic inflammation	Park and Park (2017) Safarpour and Jabbari (2018) Yuan et al. (2009)
*Opioids*							
	µ-Receptor agonist, norepinephrine and serotonin reuptake inhibitor	Tramadol		Weak, 2nd line	*4.7 (3.6–6.7)* 3.1–6.4	Also against inflammatory pain; rapid onset	Duehmke et al. (2017) Zin et al. (2008)
	µ-Receptor antagonist, noradrenaline reuptake inhibitor	Tapentadol	FDA: pDN		not reported	Rapid onset	Schwartz et al. (2015) Freo et al. (2019)
Strong opioids	µ-Receptor agonist κ-Receptor antagonist	Morphine		Weak, 3rd line	*4.3 (3.4–5.8)*	Rapid onset	Cooper et al. (2017)
	µ-Receptor agonist	Oxycodone		Weak, 3rd line	*4.3 (3.4–5.8*)	Rapid onset	Gimbel et al. (2003) Watson et al. (2003)
*Cannabinoids*							
	CB1 receptor agonists	Nabiximol	*NP*	Weak against	Not reported		Karst et al. (2003) Meng et al. (2017) Wallace et al. (2015)

*NP* neuropathic pain, *pDN* painful diabetic neuropathy, *TN* trigeminal neuralgia

^a^License according to *EMA*, if licensed by the FDA for the indication pDN = FDA: pDN

^b^NeuPSIG GRADE recommendations for NP in general (Finnerup et al. 2015)

^c^For NP in general: *Finnerup et al. *(2015) (95% CI in brackets), for the indication pDN if reported: Pop-Busui et al. (2017)

^d^Additional benefits adapted from Baron et al. (2010)

Although NP is no longer considered chronic intractable pain, meta-analyses and systematic reviews on NP indicate that only a minority of patients have an adequate response to drug therapy (Baron et al. 2010; Tan et al. 2010; Finnerup et al. 2015; Bril et al. 2011; Moulin et al. 2014; Scholz et al. 2019). Only one-third of pDN patients experience satisfying pain relief; the same is found for other patients with NP (Jensen et al. 2009). Mostly, complete freedom of pain cannot be achieved; however, improvement of life quality, sleep, social activities and ability to work is possible, which should be addressed in patient education (Baron and Binder 2016; Finnerup et al. 2015). In clinical practice, combination pharmacotherapy is often applied, although the evidence for guiding such combinations is weak (Gilron and Jensen 2013; Chaparro et al. 2012). In individual patients, second- or third-line medications may be more effective than medications coming out as first-line treatment from clinical trials. Thus, the management of patients with pDN or NP has to be on an individual basis. Non-pharmacological approaches and a generally multidisciplinary management of NP, including physical and psychological therapy and interventional approaches, need to be considered earlier (Finnerup et al. 2015; Scholz et al. 2019; Dworkin et al. 2013; Torrance et al. 2013).

### Outlook: individualized therapy of painful diabetic neuropathy

Like in other fields of medicine, sex differences and genotype are thought to play a role in predicting treatment responses in pDN and NP (Zorina-Lichtenwalter er al 2018; Belfer and Dai 2010, van Hecke et al. 2014). Genetic factors may increase the risk of developing NP and predict the response to drugs with different mechanisms of actions (Dworkin et al. 2007; Spallone 2017). So far, these factors do not play a role in designing the individualized treatment plans for patients with pDN or NP in general.

Another approach to identify possible responder populations is stratification according to the sensory phenotype of the patients, e.g., according to their QST profile or conditioned pain modulation (CPM) (Attal et al. 2011; Baron et al. 2012; Treede 2019). Recent multicenter clustering studies using QST parameters in patients with NP of different origins identified three subgroups (sensory loss, thermal hyperalgesia, and mechanical hyperalgesia; Vollert et al. 2018). Stratifying patients according to their underlying distinct mechanisms may help to decrease NNTs (Baron et al. 2017), e.g., NNT of oxcarbazepine differed between 3.9 in the irritable and 13 in the non-irritable nociceptor phenotype (Demant et al. 2014). Clonidine significantly reduced foot pain associated with pDN in a subgroup of patients with a preserved nociceptor function screened using 0.1% capsaicin (Campbell et al. 2012). Patients with pDN with a less efficient CPM, reflecting a malfunctioning pain modulation by the monoaminergic descending pathways, showed a better efficacy of duloxetine treatment (Yarnitsky et al. 2012).

Stratification according to patient-reported questionnaires such as NPSI or painDETECT seems promising (Forstenpointner et al. 2018; Bouhassira et al. 2014). Studies analyzing patterns of sensory symptoms found that classification based on the symptom clusters was more appropriate than based on etiology (Attal et al. 2008; Baron et al. 2017). In spite of the recent progress towards an individualized mechanism-based pain therapy based on the sensory phenotype, which may reflect differences in underlying mechanisms, more research is needed before personalized medicine can reach pDN patients.

## Research agenda

Although considerable research has been devoted to uncovering mechanisms of painful diabetic neuropathy (pDN) and neuropathic pain (NP) in general, treatment options to eliminate the root causes are still lacking. The research agenda for mechanism-based treatment of NP proposed in 1990 is still valid (Max 1990): “*The identification of more effective, less toxic treatments that can be individualized to patients with particular underlying mechanisms requires several coordinated research approaches:*

1. development of drugs that correct particular pathophysiological mechanisms in animal models of neuropathic pain;
2. delineation of classifications of neuropathic pain patients that correspond to the underlying pathophysiology of their pain;
3. clinical trials designed to cull out specific subsets of patients responding to a particular treatment.”

### Pharmacological targets

Current therapeutic strategies for NP, in general, aim to reduce the excitability of neurons in the peripheral and central nervous system by modulating the activity of ion channels (e.g., application of lidocaine and capsaicin), by modulating synaptic transmission (e.g., gabapentinoids) or by mimicking and enhancing endogenous inhibitory mechanisms (e.g., tricyclic antidepressants, duloxetine, and opioids). However, there is still a large gap between theoretically effective target mechanisms and actual efficacy in clinical studies.

After the discovery of complete insensitivity to pain in patients with null mutations in the sodium channel Nav1.7 (Cox et al. 2006), both academia and industry have worked intensively on specific Nav1.7 inhibitors. Until now, there has been no successful clinical trial on pain relief due to targeting selectively Nav1.7, which demonstrates the difficulties of the translation of the even most compelling targets into therapeutics. Lacosamide has a certain preference for Nav1.7, but is licensed only for the treatment of epilepsy in spite of some evidence for efficacy in pDN (Rauck et al. 2007; Wymer et al. 2009).

Considering the role of immune cells and glia in the development of NP, there has been great interest in therapeutically targeting neuroinflammation. However, most of the broad array of compounds (reviewed in Mika 2008) that successfully inactivate or disrupt glial function and attenuate pain-responsive behavior in animal models of NP are inappropriate for human application. Minocycline, a commonly used second-generation tetracycline antibiotic and non-specific blocker of microglial activation, demonstrated very promising results in the reduction of NP in preclinical studies. However, the efficacy of minocycline in clinical trials was disappointing (Martinez et al. 2013; Vanelderen et al. 2015; Sumitani et al. 2016; Curtin et al. 2017). Metformin, an oral antidiabetic used to treat T2DM, reduces microglia surrounding sensory neurons in the spinal cord and blocks neuropathic hypersensitivity in male mice, but seems to worsen the situation in females (Inyang et al. 2019). Sex differences in the role of microglia in NP mechanisms (more predominant role in male rodents) have recently dampened enthusiasm for this treatment target (North et al. 2019; Sorge et al. 2015).

Targeting metabolic and inflammatory alterations in diabetic polyneuropathy (dPNP) has been promising in preclinical animal models (reviewed in Dewanjee et al. 2018, Edwards et al. 2008). The clinical translation, however, is challenging. Currently, glycemic control and improved lifestyle remain the only disease-modifying therapies for dPNP. Benfotiamine, targeting the hexosamine pathway (Sanchez-Ramirez et al. 2006; Stracke et al. 2008), actovegin, improving tissue glucose and oxygen uptake (Ziegler et al. 2009) and the antioxidant alpha-lipoic acid (Papanas and Ziegler 2014; Ziegler et al. 2006; Ametov et al. 2003) are some of the very few strategies that lead to pain reduction in pDN patients in clinical trials among pathogenesis-oriented treatment of dPNP. However, none of them is currently approved and most of the strategies that succeeded in preclinical studies were either not tested in humans or failed in clinical trials (Dewanjee et al. 2018; Chalk et al. 2007; Grewal et al. 2016). Failure has been attributed to trial design and the inability to reach therapeutic plasma levels without toxic side effects. However, there is still extensive preclinical research on further mechanisms and new targets ongoing.

For the management of NP in general, prospective targets include several intracellular signaling pathways (Khangura et al. 2019). For dPNP and pDN in particular, recent strategies have also addressed modulating well-known targets with new pharmaceuticals, e.g., lacosamide, fulranumab and mirogabalin (Wymer et al. 2009; Rauck et al. 2007; Wang et al. 2014; Vinik et al. 2014).

### Preclinical models

One key factor for the successful clinical translation is choosing the adequate preclinical animal models of the disease and adequate outcome measures (Gregory et al. 2013; St John Smith 2018). The most frequently used rodent models look at the effects of partial mechanical nerve damage using reflex measures of stimulus-evoked pain; these models exhibit a similar sensory phenotype as peripheral nerve injury in humans (Gierthmuhlen et al. 2012), but bear poor resemblance to the clinical appearance of most NP patients.

Models that try to resemble human pDN (Gao and Zheng 2014) include high-dose streptozotocin that is toxic to pancreatic insulin-secreting β-cells and genetically modified strains are used to mimic the metabolic phenotype of T1DM. There are genetic and diet-induced animal models available to investigate NP in T2DM. However, currently, no single rodent model accurately mimics human pDN (O’Brien et al. 2014).

Moreover, the difference in pain assessment is a major problem: patients with NP primarily suffer from ongoing pain that is independent of external stimuli that is hard to measure in rodents (Tappe-Theodor and Kuner 2014). Current approaches assess pain-induced changes in gait (dynamic weight-bearing), housekeeping (burrowing), exploratory behavior (elevated plus maze, open field) or learned helplessness (forced swim test, tail suspension test).

Most knowledge about mechanisms of dPNP, pDN, and NP was gained in genetically homogenous male rodents, while patients vary in sex, ethnicity and genetic background, age, and duration of the underlying disease. To make future preclinical studies more predictive for the clinical situation, several design parameters should be expanded: (1) the wide variety of potential underlying lesions or diseases of NP suggests that a variety of models should be studied. (2) Multiple endpoints should be assessed including measures of both evoked and ongoing pain. (3) Comorbidities and quality of life should be assessed. (4) Animals with genetic variability should be used, e.g., outbred strains. (5) Studies should be done in male and female animals.

### Clinical trial design

Another key factor for a better clinical translation of preclinical studies is an improvement in clinical trial design. Compared with fields like oncology or infectious diseases, clinical trials on treating pain conditions can be quite primitive in assuming that all pain is one and the same. In the presence of evidence for multiple distinct mechanisms of pain generation, it is necessary to shift paradigms towards more specific mechanism-based indications. Clinical practice is moving towards individualized pain management, so it is advisable for the pharmaceutical industry to move in the same direction and supply the necessary specific analgesics. The European Medicines Agency published guidelines on how to conduct trials for the development of pharmaceutics for pain therapy. This guideline encourages stratification according to the phenotype (QST profile or CPM, including assessment of stimulus-evoked pain) of the patients as a first step to identify possible responder populations for existing or future medication (Cruccu and Trunini 2009; Attal et al. 2011; Baron et al. 2012; Baron et al 2017; Treede 2019). As in preclinical studies, clinical trials should include participants of both sexes, with appropriate precautions for women of childbearing age. After all, women are more frequently affected by NP than men. Finally, pain intensity as a primary endpoint should be replaced by pain severity, which is a combination of pain intensity, distress, and functional impairment (Treede et al. 2019).

## Conclusion

Treatment of NP, in general, and pDN, in particular, is challenging. Treatment of the underlying lesion or disease has been stated as a goal of NP management. This may sound manageable for dPNP via glycemic control, but clinical experience indicates that this concept holds only for T1DM, but less so for T2DM. Moreover, treating the neuropathy does not always alleviate the pain. Therefore, there is a continuing need for better analgesic medications for pDN and NP. Clinically, the field moves towards individualized pain management, which necessitates a mechanism-based pharmacotherapy of pDN. For this purpose, predictors and biomarkers need to be validated for both clinical trials and clinical practice. Several candidates have already been proposed (genetics, sex, sensory phenotype), so significant progress is expected to be made over the coming years.

## Abbreviations

AGE: Advanced glycation end products
AP: Action potential
BDNF: Brain-derived neurotrophic factor
BSE: Bedside sensory examination
CCI: Chronic constriction injury
CNS: Central nervous system
CGRP: Calcitonin gene-related peptide
DM: Diabetes mellitus
dPNP: Diabetic polyneuropathy
DRG: Dorsal root ganglion
EFNS: European Federation of Neurological Societies
EMA: European Medicines Agency
FDA: U.S. Food and Drug Administration
IASP: International Association for the Study of Pain
IL: Interleukin
LTP: Long-term potentiation
MAPK: Mitogen-activated protein kinase
MGO: Methylglyoxal
MMP: Matrix metalloproteinase
NCS: Nerve conduction study
NGF: Nerve growth factor
NeuPSIG: Neuropathic Pain Special Interest Group of IASP
NMDA R: *N*-Methyl-d-aspartate receptor
NP: Neuropathic pain
pDN: Painful diabetic neuropathy
PNS: Peripheral nervous system
ROS: Reactive oxygen species
SNI: Spared nerve injury
SNL: Spinal nerve ligation
SWME: Semmes–Weinstein monofilament examination
T1DM: Type 1 diabetes mellitus
T2DM: Type 2 diabetes mellitus
TNF-alpha: Tumor necrosis factor alpha
TRP: Transient receptor potential
TRPV1: Transient receptor potential vanilloid 1
VGSC: Voltage-gated sodium channels
vWF: von Willebrand factor

## References

[CR1] Abbott CA, Garrow AP, Carrington AL, Morris J, Van Ross ER, Boulton AJ, North-West diabetes foot care s (2005). Foot ulcer risk is lower in South-Asian and African-Caribbean compared with European diabetic patients in the U.K.: the North–West diabetes foot care study. Diabetes Care.

[CR2] Abbott CA, Malik RA, van Ross ER, Kulkarni J, Boulton AJ (2011). Prevalence and characteristics of painful diabetic neuropathy in a large community-based diabetic population in the U.K. Diabetes Care.

[CR3] Ahmad J (2016). The diabetic foot. Diabetes Metab Syndr.

[CR4] Aiyer R, Barkin RL, Bhatia A (2017). Treatment of neuropathic pain with venlafaxine: a systematic review. Pain Med.

[CR501] Alam U, Jeziorska M, Petropoulos IN, Asghar O, Fadavi H, Ponirakis G, Marshall A, Tavakoli M, Boulton AJM, Efron N, Malik RA (2017). Diagnostic utility of corneal confocal microscopy and intra-epidermal nerve fibre density in diabetic neuropathy. PLoS One.

[CR5] Albers JW (2010). Effect of prior intensive insulin treatment during the Diabetes Control and Complications Trial (DCCT) on peripheral neuropathy in type 1 diabetes during the Epidemiology of Diabetes Interventions and Complications (EDIC) Study. Diabetes Care.

[CR6] Albrecht DS (2018). Neuroinflammation of the spinal cord and nerve roots in chronic radicular pain patients. Pain.

[CR8] Allegri M, Baron R, Hans G, Correa-Illanes G, Mayoral Rojals V, Mick G, Serpell M (2016). A pharmacological treatment algorithm for localized neuropathic pain. Curr Med Res Opin.

[CR9] Alleman CJ, Westerhout KY, Hensen M, Chambers C, Stoker M, Long S, van Nooten FE (2015). Humanistic and economic burden of painful diabetic peripheral neuropathy in Europe: a review of the literature. Diabetes Res Clin Pract.

[CR10] Ametov AS (2003). The sensory symptoms of diabetic polyneuropathy are improved with alpha-lipoic acid: the SYDNEY trial. Diabetes Care.

[CR12] Amir R (2006). The role of sodium channels in chronic inflammatory and neuropathic pain. J Pain.

[CR13] Andersson C, Guttorp P, Sarkka A (2016). Discovering early diabetic neuropathy from epidermal nerve fiber patterns. Stat Med.

[CR14] Andersen ST (2018). Risk factors for incident diabetic polyneuropathy in a cohort with screen-detected Type 2 diabetes followed for 13 years: ADDITION-Denmark. Diabetes Care.

[CR15] Apfel SC (2001). Positive neuropathic sensory symptoms as endpoints in diabetic neuropathy trials. J Neurol Sci.

[CR16] Archer AG, Roberts VC, Watkins PJ (1984). Blood flow patterns in painful diabetic neuropathy. Diabetologia.

[CR17] Attal N, Fermanian C, Fermanian J, Lanteri-Minet M, Alchaar H, Bouhassira D (2008). Neuropathic pain: are there distinct subtypes depending on the aetiology or anatomical lesion?. Pain.

[CR18] Attal N (2010). EFNS guidelines on the pharmacological treatment of neuropathic pain: 2010 revision. Eur J Neurol.

[CR19] Attal N (2011). Assessing symptom profiles in neuropathic pain clinical trials: can it improve outcome?. Eur J Pain.

[CR503] Attal N, Bouhassira D (2015). Pharmacotherapy of neuropathic pain: which drugs, which treatment algorithms?. Pain.

[CR20] Attal N, Bouhassira D, Baron R (2018). Diagnosis and assessment of neuropathic pain through questionnaires. Lancet Neurol.

[CR21] Backonja MM, Coe CL, Muller DA, Schell K (2008). Altered cytokine levels in the blood and cerebrospinal fluid of chronic pain patients. J Neuroimmunol.

[CR22] Backonja MM (2013). Value of quantitative sensory testing in neurological and pain disorders: NeuPSIG consensus. Pain.

[CR23] Backryd E, Lind AL, Thulin M, Larsson A, Gerdle B, Gordh T (2017). High levels of cerebrospinal fluid chemokines point to the presence of neuroinflammation in peripheral neuropathic pain: a cross-sectional study of 2 cohorts of patients compared with healthy controls. Pain.

[CR24] Balasubramanyan S, Stemkowski PL, Stebbing MJ, Smith PA (2006). Sciatic chronic constriction injury produces cell-type-specific changes in the electrophysiological properties of rat substantia gelatinosa neurons. J Neurophysiol.

[CR25] Barbosa M, Saavedra A, Severo M, Maier C, Carvalho D (2017). Validation and reliability of the portuguese version of the Michigan neuropathy screening instrument. Pain Pract.

[CR26] Baron R (2006). Mechanisms of disease: neuropathic pain—a clinical perspective. Nat Clin Pract Neurol.

[CR27] Baron R, Binder A, Wasner G (2010). Neuropathic pain: diagnosis, pathophysiological mechanisms, and treatment. Lancet Neurol.

[CR28] Baron R, Forster M, Binder A (2012). Subgrouping of patients with neuropathic pain according to pain-related sensory abnormalities: a first step to a stratified treatment approach. Lancet Neurol.

[CR29] Baron R (2017). Peripheral neuropathic pain: a mechanism-related organizing principle based on sensory profiles. Pain.

[CR30] Bastyr EJ, Price KL, Bril V, Group MS (2005). Development and validity testing of the neuropathy total symptom score-6: questionnaire for the study of sensory symptoms of diabetic peripheral neuropathy. Clin Ther.

[CR31] Bee LA, Dickenson AH (2008). Descending facilitation from the brainstem determines behavioural and neuronal hypersensitivity following nerve injury and efficacy of pregabalin. Pain.

[CR32] Beggs S, Salter MW (2007). Stereological and somatotopic analysis of the spinal microglial response to peripheral nerve injury. Brain Behav Immun.

[CR33] Belfer I, Dai F (2010). Phenotyping and genotyping neuropathic pain. Curr Pain Headache Rep.

[CR34] Bennett DL, Woods CG (2014). Painful and painless channelopathies. Lancet Neurol.

[CR35] Bernier LP, Ase AR, Seguela P (2018). P2X receptor channels in chronic pain pathways. Br J Pharmacol.

[CR36] Bierhaus A (2012). Methylglyoxal modification of Nav1.8 facilitates nociceptive neuron firing and causes hyperalgesia in diabetic neuropathy. Nat Med.

[CR37] Binder A (2016). Human surrogate models of neuropathic pain: validity and limitations. Pain.

[CR38] Blesneac I (2018). Rare NaV1.7 variants associated with painful diabetic peripheral neuropathy. Pain.

[CR40] Borsook D, Kussman BD, George E, Becerra LR, Burke DW (2013). Surgically induced neuropathic pain: understanding the perioperative process. Ann Surg.

[CR41] Bosma RL (2018). Brain dynamics and temporal summation of pain predicts neuropathic pain relief from ketamine infusion. Anesthesiology.

[CR43] Bouhassira D, Attal N (2011). Diagnosis and assessment of neuropathic pain: the saga of clinical tools. Pain.

[CR44] Bouhassira D, Attal N, Fermanian J, Alchaar H, Gautron M, Masquelier E, Rostaing S, Lanteri-Minet M, Collin E, Grisart J, Boureau F (2004). Development and validation of the neuropathic pain symptom. Inventory Pain.

[CR45] Bouhassira D (2005). Comparison of pain syndromes associated with nervous or somatic lesions and development of a new neuropathic pain diagnostic questionnaire (DN4). Pain.

[CR46] Bouhassira D, Lantéri-Minet M, Attal N, Laurent B, Touboul C (2008). Prevalence of chronic pain wih neuropathic characteristics in the general population. Pain.

[CR47] Bouhassira D, Letanoux M, Hartemann A (2013). Chronic pain with neuropathic characteristics in diabetic patients: a French cross-sectional study. PLoS ONE.

[CR48] Bouhassira D (2014). Neuropathic pain phenotyping as a predictor of treatment response in painful diabetic neuropathy: data from the randomized, double-blind, COMBO-DN study. Pain.

[CR49] Boulton AJ (2008). The diabetic foot—an update. Foot Ankle Surg.

[CR50] Bril V, Perkins BA (2002). Validation of the Toronto Clinical Scoring system for diabetic polyneuropathy. Diabetes Care.

[CR51] Bril V, Tomioka S, Buchanan RA, Perkins BA, mTCNS Study Group (2009). Reliability and validity of the modified Toronto Clinical Neuropathy Score in diabetic sensorimotor polyneuropathy. Diabet Med.

[CR52] Bril V (2011). Evidence-based guideline: treatment of painful diabetic neuropathy: report of the American Academy of Neurology, the American Association of Neuromuscular and Electrodiagnostic Medicine, and the American Academy of Physical Medicine and Rehabilitation. Neurology.

[CR53] Brown JJ, Pribesh SL, Baskette KG, Vinik AI, Colberg SR (2017). A comparison of screening tools for the early detection of peripheral neuropathy in adults with and without type 2 diabetes. J Diabetes Res.

[CR54] Burke D, Fullen BM, Stokes D, Lennon O (2017). Neuropathic pain prevalence following spinal cord injury: a systematic review and meta-analysis. Eur J Pain.

[CR55] Busserolles J, Tsantoulas C, Eschalier A, Lopez Garcia JA (2016). Potassium channels in neuropathic pain: advances, challenges, and emerging ideas. Pain.

[CR56] Callaghan BC, Cheng HLT, Stables CL, Smith AL, Feldman EL (2012). Diabetic neuropathy: clinical manifestations and current treatments. Lancet Neurol.

[CR57] Callaghan BC, Little AA, Feldman EL, Hughes RAC (2012). Enhanced glucose control for preventing and treating diabetic neuropathy. Cochrane Database Syst Rev.

[CR58] Calles-Escandon J (2010). Effect of intensive compared with standard glycemia treatment strategies on mortality by baseline subgroup characteristics: the Action to Control Cardiovascular Risk in Diabetes (ACCORD) trial. Diabetes Care.

[CR59] Cameron NE, Eaton SE, Cotter MA, Tesfaye S (2001). Vascular factors and metabolic interactions in the pathogenesis of diabetic neuropathy. Diabetologia.

[CR60] Campbell JN, Meyer RA (2006). Mechanisms of neuropathic pain. Neuron.

[CR62] Campbell CM (2012). Randomized control trial of topical clonidine for treatment of painful diabetic neuropathy. Pain.

[CR63] Caselli A, Rich J, Hanane T, Uccioli L, Veves A (2003). Role of C-nociceptive fibers in the nerve axon reflex-related vasodilation in diabetes. Neurology.

[CR64] Caterina MJ, Schumacher MA, Tominaga M, Rosen TA, Levine JD, Julius D (1997). The capsaicin receptor: a heat-activated ion channel in the pain pathway. Nature.

[CR65] Challa SR (2015). Surgical animal models of neuropathic pain: Pros and Cons. Int J Neurosci.

[CR66] Chalk C, Benstead TJ, Moore F (2007). Aldose reductase inhibitors for the treatment of diabetic polyneuropathy. Cochrane Database Syst Rev.

[CR67] Chaparro LE, Wiffen PJ, Moore RA, Gilron I (2012). Combination pharmacotherapy for the treatment of neuropathic pain in adults. Cochrane Database Syst Rev.

[CR68] Chaplan SR (2003). Neuronal hyperpolarization-activated pacemaker channels drive neuropathic pain. J Neurosci.

[CR69] Cheliout-Heraut F (2005). Exploration of small fibers for testing diabetic neuropathies. Jt Bone Spine.

[CR70] Chen L, Huang LY (1992). Protein kinase C reduces Mg^2+^ block of NMDA-receptor channels as a mechanism of modulation. Nature.

[CR71] Chen J (2018). The alpha2delta-1-NMDA receptor complex is critically involved in neuropathic pain development and gabapentin therapeutic actions. Cell Rep.

[CR72] Cheng HT (2013). Increased axonal regeneration and swellings in intraepidermal nerve fibers characterize painful phenotypes of diabetic neuropathy. J Pain.

[CR74] Choi SR (2017). Spinal D-serine increases PKC-dependent GluN1 phosphorylation contributing to the sigma-1 receptor-induced development of mechanical allodynia in a mouse model of neuropathic pain. J Pain.

[CR75] Clark AK, Malcangio M (2012). Microglial signalling mechanisms: cathepsin S and fractalkine. Exp Neurol.

[CR76] Clark AK, Malcangio M (2014). Fractalkine/CX3CR1 signaling during neuropathic pain. Front Cell Neurosci.

[CR77] Colleoni M, Sacerdote P (2010). Murine models of human neuropathic pain. Biochem Biophys Acta.

[CR78] Colloca L (2017). Neuropathic pain. Nat Rev Dis Primers.

[CR79] Collins SL, Moore RA, McQuayHj WP (2000). Antidepressants and anticonvulsants for diabetic neuropathy and postherpetic neuralgia: a quantitative systematic review. J Pain Symptom Manag.

[CR80] Cooper TE (2017). Morphine for chronic neuropathic pain in adults. Cochrane Database Syst Rev.

[CR81] Cornblath DR, Chaudhry V, Carter K, Lee D, Seysedadr M, Miernicki M, Joh T (1999). Total neuropathy score: validation and reliability study. Neurology.

[CR82] Costigan M, Scholz J, Woolf CJ (2009). Neuropathic pain: a maladaptive response of the nervous system to damage. Annu Rev Neurosci.

[CR83] Coull JA (2005). BDNF from microglia causes the shift in neuronal anion gradient underlying neuropathic pain. Nature.

[CR84] Coward K (2000). Immunolocalization of SNS/PN3 and NaN/SNS2 sodium channels in human pain states. Pain.

[CR85] Cox JJ (2006). An SCN9A channelopathy causes congenital inability to experience pain. Nature.

[CR86] Crawford B, Bouhassira D, Wong A, Dukes E (2008). Conceptual adequacy of the neuropathic pain symptom inventory in six countries. Health Qual Life Outcomes.

[CR87] Cruccu G, Truini A (2009). Sensory profiles: a new strategy for selecting patients in treatment trials for neuropathic pain. Pain.

[CR88] Cruccu G, Truini A (2017). A review of neuropathic pain: from guidelines to clinical practice. Pain Ther.

[CR89] Cruccu G (2008). Recommendations for the clinical use of somatosensory-evoked potentials. Clin Neurophysiol.

[CR90] Cruccu G (2010). EFNS guidelines on neuropathic pain assessment: revised 2009. Eur J Neurol.

[CR91] Cruccu G (2016). Trigeminal neuralgia: new classification and diagnostic grading for practice and research. Neurology.

[CR92] Cummins TR, Sheets PL, Waxman SG (2007). The roles of sodium channels in nociception: implications for mechanisms of pain. Pain.

[CR93] Curtin CM, Kenney D, Suarez P, Hentz VR, Hernandez-Boussard T, Mackey S, Carroll IR (2017). A double-blind placebo randomized controlled trial of minocycline to reduce pain after carpal tunnel and trigger finger release. J Hand Surg Am.

[CR94] Davis KD (2017). Brain imaging tests for chronic pain: medical, legal and ethical issues and recommendations. Nat Rev Neurol.

[CR96] De Keyser R, van den Broeke EN, Courtin A, Dufour A, Mouraux A (2018). Event-related brain potentials elicited by high-speed cooling of the skin: a robust and non-painful method to assess the spinothalamic system in humans. Clin Neurophysiol.

[CR97] Delpont B, Blanc C, Osseby GV, Hervieu-Begue M, Giroud M, Bejot Y (2018). Pain after stroke: a review. Rev Neurol (Paris).

[CR98] Demant DT (2014). The effect of oxcarbazepine in peripheral neuropathic pain depends on pain phenotype: a randomised, double-blind, placebo-controlled phenotype-stratified study. Pain.

[CR99] Deng Y, Luo L, Hu Y, Fang K, Liu J (2016). Clinical practice guidelines for the management of neuropathic pain: a systematic review. BMC Anesthesiol.

[CR101] Derry S, Rice AS, Cole P, Tan T, Moore RA (2017). Topical capsaicin (high concentration) for chronic neuropathic pain in adults. Cochrane Database Syst Rev.

[CR102] Derry S, Bell RF, Straube S, Wiffen PJ, Aldington D, Moore RA (2019). Pregabalin for neuropathic pain in adults. Cochrane Database Syst Rev.

[CR103] Devigili G (2008). The diagnostic criteria for small fibre neuropathy: from symptoms to neuropathology. Brain.

[CR104] Devor M (2009). Ectopic discharge in Abeta afferents as a source of neuropathic pain. Exp Brain Res.

[CR105] Dewanjee S (2018). Molecular mechanism of diabetic neuropathy and its pharmacotherapeutic targets. Eur J Pharmacol.

[CR106] Di Stefano G (2017). Diagnostic accuracy of laser-evoked potentials in diabetic neuropathy. Pain.

[CR107] Dib-Hajj SD, Cummins TR, Black JA, Waxman SG (2010). Sodium channels in normal and pathological pain. Annu Rev Neurosci.

[CR109] Doth AH, Hansson PT, Jensen MP, Taylor RS (2010). The burden of neuropathic pain: a systematic review and meta-analysis of health utilities. Pain.

[CR110] Drdla-Schutting R, Benrath J, Wunderbaldinger G, Sandkuhler J (2012). Erasure of a spinal memory trace of pain by a brief, high-dose opioid administration. Science (New York, NY).

[CR111] Dull MM (2019). Methylglyoxal causes pain and hyperalgesia in human through C-fiber activation. Pain.

[CR112] Duehmke RM, Derry S, Wiffen PJ, Bell RF, Aldington D, Moore RA (2017). Tramadol for neuropathic pain in adults. Cochrane Database Syst Rev.

[CR113] Dworkin RH (2007). Pharmacologic management of neuropathic pain: evidence-based recommendations. Pain.

[CR114] Dworkin RH (2010). Recommendations for the pharmacological management of neuropathic pain: an overview and literature update. Mayo Clin Proc.

[CR115] Dworkin RH (2013). Interventional management of neuropathic pain: NeuPSIG recommendations. Pain.

[CR116] Dyck PJ (1988). Detection, characterization, and staging of polyneuropathy: assessed in diabetics. Muscle Nerve.

[CR117] Dyck PJ (1980). Human diabetic endoneurial sorbitol, fructose, and myo-inositol related to sural nerve morphometry. Ann Neurol.

[CR118] Dyck PJ, Karnes JL, O'Brien PC, Litchy WJ, Low PA, Melton LJ (1992). The Rochester Diabetic Neuropathy Study: reassessment of tests and criteria for diagnosis and staged severity. Neurology.

[CR119] Dyck PJ (1993). The prevalence by staged severity of various types of diabetic neuropathy, retinopathy, and nephropathy in a population-based cohort: the Rochester Diabetic Neuropathy Study. Neurology.

[CR120] Dyck PJ (2010). Signs and symptoms versus nerve conduction studies to diagnose diabetic sensorimotor polyneuropathy: Cl vs NPhys trial. Muscle Nerve.

[CR121] Dyck PJ, Carter RE, Litchy WJ (2011). Modeling nerve conduction criteria for diagnosis of diabetic polyneuropathy. Muscle Nerve.

[CR122] Edwards JL, Vincent AM, Cheng HT, Feldman EL (2008). Diabetic neuropathy: mechanisms to management. Pharmacol Ther.

[CR123] Edwards RR (2016). Patient phenotyping in clinical trials of chronic pain treatments: IMMPACT recommendations. Pain.

[CR125] Ellis RJ (2010). Continued high prevalence and adverse clinical impact of human immunodeficiency virus-associated sensory neuropathy in the era of combination antiretroviral therapy: the CHARTER Study. Arch Neurol.

[CR126] England JD (2005). Distal symmetrical polyneuropathy: a definition for clinical research. A report of the American Academy of Neurology, the American Association of Electrodiagnostic Medicine, and the American Academy of Physical Medicine and Rehabilitation. Arch Phys Med Rehabil.

[CR127] Epping R, Verhagen AP, Hoebink EA, Rooker S, Scholten-Peeters GGM (2017). The diagnostic accuracy and test–retest reliability of the Dutch PainDETECT and the DN4 screening tools for neuropathic pain in patients with suspected cervical or lumbar radiculopathy. Musculoskelet Sci Pract.

[CR128] Estacion M (2008). NaV1.7 gain-of-function mutations as a continuum: A1632E displays physiological changes associated with erythromelalgia and paroxysmal extreme pain disorder mutations and produces symptoms of both disorders. J Neurosci.

[CR129] European Medicines Agency. EMA/CHMP/970057/2011: guideline on the clinical development of medicinal products intended for the treatment of pain. https://www.ema.europa.eu/docs/en_GB/document_library/Scientific_guideline/2016/12/WC500219131.pdf. Accessed 15 Sep 2019

[CR130] Ezenwa M, Yao Y, Suarez M, Zhao Z, Carrasco J, Angulo V, Shuey D, Roach K, Wang Z, Molokie R, Wilkie D (2016). (187) Normative values for quantitative sensory testing in African Americans. J Pain.

[CR131] Farooqi MA (2016). Validation of cooling detection threshold as a marker of sensorimotor polyneuropathy in type 2 diabetes. J Diabetes Complications.

[CR133] Feldman EL, Stevens MJ, Thomas PK, Brown MB, Canal N, Greene DA (1994). A practical two-step quantitative clinical and electrophysiological assessment for the diagnosis and staging of diabetic neuropathy. Diabetes Care.

[CR134] Feldman EL, Nave KA, Jensen TS, Bennett DLH (2017). New horizons in diabetic neuropathy: mechanisms, bioenergetics, and pain. Neuron.

[CR135] Feldman EL (2019). Diabetic neuropathy. Nat Rev Dis Primers.

[CR137] Fernyhough P (2015). Mitochondrial dysfunction in diabetic neuropathy: a series of unfortunate metabolic events. Curr DiabRep.

[CR138] Fernyhough P, McGavock J (2014). Mechanisms of disease: mitochondrial dysfunction in sensory neuropathy and other complications in diabetes. Handb Clin Neurol.

[CR139] Fields HL, Rowbotham M, Baron R (1998). Postherpetic neuralgia: irritable nociceptors and deafferentation. Neurobiol Dis.

[CR140] Finnerup NB, Sindrup SH, Jensen TS (2010). The evidence for pharmacological treatment of neuropathic pain. Pain.

[CR141] Finnerup NB (2013). Neuropathic pain needs systematic classification. Eur J Pain.

[CR142] Finnerup NB (2015). Pharmacotherapy for neuropathic pain in adults: a systematic review and meta-analysis. Lancet Neurol.

[CR143] Finnerup NB (2016). Neuropathic pain: an updated grading system for research and clinical practice. Pain.

[CR144] Foley PL (2013). Prevalence and natural history of pain in adults with multiple sclerosis: systematic review and meta-analysis. Pain.

[CR145] Forbes HJ, Thomas SL, Smeeth L, Clayton T, Farmer R, Bhaskaran K, Langan SM (2016). A systematic review and meta-analysis of risk factors for postherpetic neuralgia. Pain.

[CR146] Forstenpointner J, Otto J, Baron R (2018). Individualized neuropathic pain therapy based on phenotyping: are we there yet?. Pain.

[CR147] Frazier WA, Angeletti RH, Bradshaw RA (1972). Nerve growth factor and insulin. Science (New York, NY).

[CR148] Freo U, Romualdi P, Kress HG (2019). Tapentadol for neuropathic pain: a review of clinical studies. J Pain Res.

[CR149] Freynhagen R, Strojek K, Griesing T, Whalen E, Balkenohl M (2005). Efficacy of pregabalin in neuropathic pain evaluated in a 12-week, randomised, double-blind, multicentre, placebo-controlled trial of flexible- and fixed-dose regimens. Pain.

[CR150] Freynhagen R, Baron R, Gockel U, Tolle TR (2006). painDETECT: a new screening questionnaire to identify neuropathic components in patients with back pain. Curr Med Res Opin.

[CR151] Freynhagen R (2019). Current understanding of the mixed pain concept: a brief narrative review. Curr Med Res Opin.

[CR152] Fukuoka M, Sakurai K, Ohta T, Kiyoki M, Katayama I (2001). Tacalcitol, an active vitamin D3, induces nerve growth factor production in human epidermal keratinocytes. Skin Pharmacol Appl Skin Physiol.

[CR153] Fullerton B, Jeitler K, Seitz M, Horvath K, Berghold A, Siebenhofer A (2014). Intensive glucose control versus conventional glucose control for type 1 diabetes mellitus. Cochrane Database Syst Rev.

[CR154] Gallagher HC, Gallagher RM, Butler M, Buggy DJ, Henman MC (2015). Venlafaxine for neuropathic pain in adults. Cochrane Database Syst Rev.

[CR155] Gao F, Zheng ZM (2014). Animal models of diabetic neuropathic pain. Exp Clin Endocrinol Diabetes.

[CR156] Gasparotti R, Padua L, Briani C, Lauria G (2017). New technologies for the assessment of neuropathies. Nat Rev Neurol.

[CR157] German National Disease Management Guideline for Diabetic Neuropathy: Bundesärztekammer (BÄK), Kassenärztliche Bundesvereinigung (KBV), Arbeitsgemeinschaft der Wissenschaftli-chen Medizinischen Fachgesellschaften (AWMF). Nationale VersorgungsLeitlinie Neuropathie bei Diabetes im Erwachsenenalter—Langfassung, 1. Auflage. Version 5. 2011. www.dm-neuropathie.versorgungsleitlinien.de. Accessed 17 Sep 2019. 10.6101/AZQ/000302

[CR158] Gibbons CH (2017). Treatment-induced neuropathy of diabetes. Curr Diab Rep.

[CR159] Gibbons CH (2017). Treatment induced neuropathy of diabetes—long term implications in type 1 diabetes. J Diabetes Complications.

[CR160] Gibbons CH, Freeman R (2015). Treatment-induced neuropathy of diabetes: an acute, iatrogenic complication of diabetes. Brain.

[CR161] Gierthmuhlen J, Baron R (2016). Neuropathic pain. Semin Neurol.

[CR162] Gierthmühlen J, Maier C, Baron R, Tölle T, Treede RD, Birbaumer N, Huge V, Koroschetz J, Krumova EK, Lauchart M, Maihöfner C, Richter H, Westermann A, DFNS Study Group (2012). Sensory signs in complex regional pain syndrome and peripheral nerve injury. Pain.

[CR163] Gilron I, Jensen TS, Dickenson AH (2013). Combination pharmacotherapy for management of chronic pain: from bench to bedside. Lancet Neurol.

[CR164] Gimbel JS, Richards P, Portenoy RK (2003). Controlled-release oxycodone for pain in diabetic neuropathy: a randomized controlled trial. Neurology.

[CR165] Gold MS, Gebhart GF (2010). Nociceptor sensitization in pain pathogenesis. Nat Med.

[CR166] Gold MS, Reichling DB, Shuster MJ, Levine JD (1996). Hyperalgesic agents increase a tetrodotoxin-resistant Na+ current in nociceptors. Proc Natl Acad Sci USA.

[CR167] Gonzalez-Duarte A, Lem-Carrillo M, Guerrero-Torres L (2016). Normative values of quantitative sensory testing in Hispanic Latino population. Brain Behav.

[CR168] Goodman CW, Brett AS (2017). Gabapentin and Pregabalin for pain—is increased prescribing a cause for concern?. N Engl J Med.

[CR169] Gore M, Brandenburg NA, Dukes E, Hoffman DL, Tai KS, Stacey B (2005). Pain severity in diabetic peripheral neuropathy is associated with patient functioning, symptom levels of anxiety and depression, and sleep. J Pain Symptom Manag.

[CR170] Gracely RH, Lynch SA, Bennett GJ (1992). Painful neuropathy: altered central processing maintained dynamically by peripheral input. Pain.

[CR171] Granovsky Y (2013). Conditioned pain modulation: a predictor for development and treatment of neuropathic pain. Curr Pain Headache Rep.

[CR172] Granovsky Y, Nahman-Averbuch H, Khamaisi M, Granot M (2017). Efficient conditioned pain modulation despite pain persistence in painful diabetic neuropathy. Pain Rep.

[CR173] Gregory NS, Harris AL, Robinson CR, Dougherty PM, Fuchs PN, Sluka KA (2013). An overview of animal models of pain: disease models and outcome measures. J Pain.

[CR175] Grewal AS, Bhardwaj S, Pandita D, Lather V, Sekhon BS (2016). Updates on aldose reductase inhibitors for management of diabetic complications and non-diabetic diseases. Mini Rev Med Chem.

[CR177] Grote CW, Wright DE (2016). A role for insulin in diabetic neuropathy. Front Neurosci.

[CR178] Gruber-Schoffnegger D, Drdla-Schutting R, Honigsperger C, Wunderbaldinger G, Gassner M, Sandkuhler J (2013). Induction of thermal hyperalgesia and synaptic long-term potentiation in the spinal cord lamina I by TNF-alpha and IL-1beta is mediated by glial cells. J Neurosci.

[CR500] Haanpaa ML, Backonja MM, Bennett MI, Bouhassira D, Cruccu G, Hansson PT, Jensen TS, Kauppila T, Rice ASC, Smith BH, Treede RD, Baron R (2009). Assessment of neuropathic pain in primary care. Am J Med.

[CR179] Haanpaa M (2011). NeuPSIG guidelines on neuropathic pain assessment. Pain.

[CR180] Hains BC, Waxman SG (2007). Sodium channel expression and the molecular pathophysiology of pain after SCI. Prog Brain Res.

[CR181] Hameed S (2019). Nav1.7 and Nav1.8: role in the pathophysiology of pain. Mol Pain.

[CR182] Hansson P (2002). Neuropathic pain: clinical characteristics and diagnostic workup. Eur J Pain.

[CR183] Haroutounian S, Nikolajsen L, Bendtsen TF, Finnerup NB, Kristensen AD, Hasselstrom JB, Jensen TS (2014). Primary afferent input critical for maintaining spontaneous pain in peripheral neuropathy. Pain.

[CR184] Hatch MN, Cushing TR, Carlson GD, Chang EY (2018). Neuropathic pain and SCI: identification and treatment strategies in the 21st century. J Neurol Sci.

[CR185] Hearn L, Derry S, Phillips T, Moore RA, Wiffen PJ (2014). Imipramine for neuropathic pain in adults. Cochrane Database Syst Rev.

[CR186] Hearn L, Moore RA, Derry S, Wiffen PJ, Phillips T (2014). Desipramine for neuropathic pain in adults. Cochrane Database Syst Rev.

[CR187] Hebert HL, Veluchamy A, Torrance N, Smith BH (2017). Risk factors for neuropathic pain in diabetes mellitus. Pain.

[CR189] Herder C (2017). Proinflammatory cytokines predict the incidence and progression of distal sensorimotor polyneuropathy: KORA F4/FF4 study. Diabetes Care.

[CR190] Hildebrand ME (2016). Potentiation of synaptic GluN2B NMDAR currents by Fyn kinase is gated through BDNF-mediated disinhibition in spinal pain processing. Cell Rep.

[CR191] Hirayasu K (2018). Difference in normal limit values of nerve conduction parameters between Westerners and Japanese people might need to be considered when diagnosing diabetic polyneuropathy using a Point-of-Care Sural Nerve Conduction Device (NC-stat(R)/DPNCheck). J Diabetes Investig.

[CR192] Hoeijmakers JG, Faber CG, Merkies IS, Waxman SG (2015). Painful peripheral neuropathy and sodium channel mutations. Neurosci Lett.

[CR193] Hoffmann T, Kistner K, Carr RW, Nassar MA, Reeh PW, Weidner C (2017). Reduced excitability and impaired nociception in peripheral unmyelinated fibers from Nav1.9-null mice. Pain.

[CR194] Holiner I (2013). Validity of the neurological examination in diagnosing diabetic peripheral neuropathy. Pediatr Neurol.

[CR195] Hsieh ST (2010). Pathology and functional diagnosis of small-fiber painful neuropathy. Acta Neurol Taiwan.

[CR196] Huang Q, Chen Y, Gong N, Wang YX (2016). Methylglyoxal mediates streptozotocin-induced diabetic neuropathic pain via activation of the peripheral TRPA1 and Nav1.8 channels. Metab Clin Exp.

[CR198] Hwang YT, Davies G (2016). 'Insulin neuritis' to 'treatment-induced neuropathy of diabetes': new name, same mystery. Pract Neurol.

[CR199] IASP (2011) Part III: Pain terms. A current list with definitions and notes on usage. International Association for the Study of Pain (IASP). Pain terms. https://www.iasp-pain.org/terminology. Accessed 15 Mar 2019

[CR200] IDF Diabetes Atlas, 8th ed. https://www.diabetesatlas.org/across-the-globe.html. Accessed 30 Oct 2019

[CR201] Inoue K (2017). Purinergic signaling in microglia in the pathogenesis of neuropathic pain. Proc Jpn Acad Ser B Phys Biol Sci.

[CR202] Inoue K, Tsuda M (2018). Microglia in neuropathic pain: cellular and molecular mechanisms and therapeutic potential. Nat Rev Neurosci.

[CR203] Inyang KE, Szabo-Pardi T, Wentworth E, McDougal TA, Dussor G, Burton MD, Price TJ (2019). The antidiabetic drug metformin prevents and reverses neuropathic pain and spinal cord microglial activation in male but not female mice. Pharmacol Res.

[CR204] Ismail-Beigi F (2010). Effect of intensive treatment of hyperglycaemia on microvascular outcomes in type 2 diabetes: an analysis of the ACCORD randomised trial. Lancet.

[CR205] Jaggi AS, Jain V, Singh N (2011). Animal models of neuropathic pain. Fundam Clin Pharmacol.

[CR206] Jain R, Jain S, Raison CL, Maletic V (2011). Painful diabetic neuropathy is more than pain alone: examining the role of anxiety and depression as mediators and complicators. Curr Diab Rep.

[CR208] Jay GW, Barkin RL (2014). Neuropathic pain: etiology, pathophysiology, mechanisms, and evaluations. Dis Mon.

[CR209] Jensen TS, Finnerup NB (2014). Allodynia and hyperalgesia in neuropathic pain: clinical manifestations and mechanisms. Lancet Neurol.

[CR210] Jensen TS, Bach FW, Kastrup J, Dejgaard A, Brennum J (1991). Vibratory and thermal thresholds in diabetics with and without clinical neuropathy. Acta Neurol Scand.

[CR211] Jensen TS, Madsen CS, Finnerup NB (2009). Pharmacology and treatment of neuropathic pains. Curr Opin Neurol.

[CR212] Jensen TS, Baron R, Haanpaa M, Kalso E, Loeser JD, Rice AS, Treede RD (2011). A new definition of neuropathic pain. Pain.

[CR213] Ji RR, Kohno T, Moore KA, Woolf CJ (2003). Central sensitization and LTP: do pain and memory share similar mechanisms?. Trends Neurosci.

[CR214] Ji RR, Berta T, Nedergaard M (2013). Glia and pain: is chronic pain a gliopathy?. Pain.

[CR215] Ji RR, Nackley A, Huh Y, Terrando N, Maixner W (2018). Neuroinflammation and central sensitization in chronic and widespread pain. Anesthesiology.

[CR216] Jiang B, Zhang Y, Zhao J, She C, Zhou X, Dong Q, Wang P (2017). Effects of localized X-ray irradiation on peripheral nerve regeneration in transected sciatic nerve in rats. Radiat Res.

[CR217] Jin SX, Zhuang ZY, Woolf CJ, Ji RR (2003). p38 mitogen-activated protein kinase is activated after a spinal nerve ligation in spinal cord microglia and dorsal root ganglion neurons and contributes to the generation of neuropathic pain. J Neurosci.

[CR218] Karst M, Salim K, Burstein S, Conrad I, Hoy L, Schneider U (2003). Analgesic effect of the synthetic cannabinoid CT-3 on chronic neuropathic pain: a randomized controlled trial. JAMA.

[CR219] Kavitha KV, Tiwari S, Purandare VB, Khedkar S, Bhosale SS, Unnikrishnan AG (2014). Choice of wound care in diabetic foot ulcer: a practical approach. World J Diabetes.

[CR220] Keller AF, Beggs S, Salter MW, De Koninck Y (2007). Transformation of the output of spinal lamina I neurons after nerve injury and microglia stimulation underlying neuropathic pain. Mol Pain.

[CR221] Kennedy DL, Kemp HI, Ridout D, Yarnitsky D, Rice AS (2016). Reliability of conditioned pain modulation: a systematic review. Pain.

[CR222] Khangura RK, Sharma J, Bali A, Singh N, Jaggi AS (2019). An integrated review on new targets in the treatment of neuropathic pain. Korean J Physiol Pharmacol.

[CR223] Kim SK (2016). Cortical astrocytes rewire somatosensory cortical circuits for peripheral neuropathic pain. J Clin Investig.

[CR225] Klein T, Magerl W, Hopf HC, Sandkuhler J, Treede RD (2004). Perceptual correlates of nociceptive long-term potentiation and long-term depression in humans. J Neurosci.

[CR226] Klein T, Magerl W, Rolke R, Treede RD (2005). Human surrogate models of neuropathic pain. Pain.

[CR227] Kobayashi M, Zochodne DW (2018). Diabetic neuropathy and the sensory neuron: new aspects of pathogenesis and their treatment implications. J Diabetes Investig.

[CR228] Kopf S (2018). Deep phenotyping neuropathy: an underestimated complication in patients with pre-diabetes and type 2 diabetes associated with albuminuria. Diabetes Res Clin Pract.

[CR229] Kosek E (2016). Do we need a third mechanistic descriptor for chronic pain states?. Pain.

[CR230] Kotani N (2004). Cerebrospinal fluid interleukin 8 concentrations and the subsequent development of postherpetic neuralgia. Am J Med.

[CR231] Krause SJ, Backonja MM (2003). Development of a neuropathic pain questionnaire. Clin J Pain.

[CR232] Krishnan ST, Rayman G (2004). The LDIflare: a novel test of C-fiber function demonstrates early neuropathy in type 2 diabetes. Diabetes Care.

[CR233] Lacagnina MJ, Watkins LR, Grace PM (2018). Toll-like receptors and their role in persistent pain. Pharmacol Ther.

[CR234] La Cesa S (2015). How to diagnose neuropathic pain? The contribution from clinical examination, pain questionnaires and diagnostic tests. Neurol Sci.

[CR235] Lai J, Hunter JC, Porreca F (2003). The role of voltage-gated sodium channels in neuropathic pain. Curr Opin Neurobiol.

[CR236] Lai J, Porreca F, Hunter JC, Gold MS (2004). Voltage-gated sodium channels and hyperalgesia. Annu Rev Pharmacol Toxicol.

[CR237] Laiteerapong N, Ham SA, Gao Y, Moffet HH, Liu JY, Huang ES, Karter AJ (2019). The legacy effect in Type 2 diabetes: impact of early glycemic control on future complications (the diabetes and aging study). Diabetes Care.

[CR238] Latremoliere A, Woolf CJ (2009). Central sensitization: a generator of pain hypersensitivity by central neural plasticity. J Pain.

[CR239] Lauria G (2010). European Federation of Neurological Societies/Peripheral Nerve Society Guideline on the use of skin biopsy in the diagnosis of small fiber neuropathy. Report of a joint task force of the European Federation of Neurological Societies and the Peripheral Nerve Society. Eur J Neurol.

[CR240] Leone C (2019). Cooling the skin for assessing small-fibre function. Pain.

[CR241] Lenz FA, Kwan HC, Martin R, Tasker R, Richardson RT, Dostrovsky JO (1994). Characteristics of somatotopic organization and spontaneous neuronal activity in the region of the thalamic principal sensory nucleus in patients with spinal cord transection. J Neurophysiol.

[CR244] Liyanage PL, Lekamwasam S, Weerarathna TP (2012). Validity of the Diabetic Neuropathy Score and Diabetic Neuropathy Examination score as screening tools for the detection of distal symmetrical diabetic neuropathy. J Diabetes.

[CR245] Loeser JD, Treede RD (2008). The Kyoto protocol of IASP basic pain terminology. Pain.

[CR246] Love S (1983). An experimental study of peripheral nerve regeneration after x-irradiation. Brain.

[CR247] Low PA, Singer W (2015). Treatment-induced neuropathy of diabetes: an energy crisis?. Brain.

[CR248] Lu WY, Xiong ZG, Lei S, Orser BA, Dudek E, Browning MD, MacDonald JF (1999). G-protein-coupled receptors act via protein kinase C and Src to regulate NMDA receptors. Nat Neurosci.

[CR249] Lunn MP, Hughes RA, Wiffen PJ (2014). Duloxetine for treating painful neuropathy, chronic pain or fibromyalgia. Cochrane Database Syst Rev.

[CR250] Luo ZD, Chaplan SR, Higuera ES, Sorkin LS, Stauderman KA, Williams ME, Yaksh TL (2001). Upregulation of dorsal root ganglion (alpha)2(delta) calcium channel subunit and its correlation with allodynia in spinal nerve-injured rats. J Neurosci.

[CR251] Lucchetta M, Pazzaglia C, Padua L, Briani C (2011). Exploring neuropathic symptoms in a large cohort of Italian patients with different peripheral nervous system diseases. Neurol Sci.

[CR252] Magerl W, Krumova EK, Baron R, Tolle T, Treede RD, Maier C (2010). Reference data for quantitative sensory testing (QST): refined stratification for age and a novel method for statistical comparison of group data. Pain.

[CR254] Magrinelli F (2015). The association between serum cytokines and damage to large and small nerve fibers in diabetic peripheral neuropathy. J Diabetes Res.

[CR255] Malik RA (2005). Sural nerve pathology in diabetic patients with minimal but progressive neuropathy. Diabetologia.

[CR256] Mallick-Searle T, Snodgrass B, Brant JM (2016). Postherpetic neuralgia: epidemiology, pathophysiology, and pain management pharmacology. J Multidiscip Healthc.

[CR257] Manchikanti L, Singh V (2004). Managing phantom pain. Pain Phys.

[CR258] Marchand F, Perretti M, McMahon SB (2005). Role of the immune system in chronic pain. Nat Rev Neurosci.

[CR259] Margolis RB, Tait RC, Krause SJ (1986). A rating system for use with patient pain drawings. Pain.

[CR261] Martin MM (1953). Diabetic neuropathy; a clinical study of 150 cases. Brain.

[CR262] Martinez V (2013). The efficacy of a glial inhibitor, minocycline, for preventing persistent pain after lumbar discectomy: a randomized, double-blind, controlled study. Pain.

[CR263] Mathieson S, Maher CG, Terwee CB, Folly de Campos T, Lin CW (2015). Neuropathic pain screening questionnaires have limited measurement properties. A systematic review. J Clin Epidemiol.

[CR264] Max MB (1990). Towards physiologically based treatment of patients with neuropathic pain. Pain.

[CR265] May A (2008). Chronic pain may change the structure of the brain. Pain.

[CR267] McMahon SB, Malcangio M (2009). Current challenges in glia-pain biology. Neuron.

[CR268] Meacham K, Shepherd A, Mohapatra DP, Haroutounian S (2017). Neuropathic pain: central vs. peripheral mechanisms. Curr Pain Headache Rep.

[CR269] Mehra S, Tavakoli M, Kallinikos PA, Efron N, Boulton AJ, Augustine T, Malik RA (2007). Corneal confocal microscopy detects early nerve regeneration after pancreas transplantation in patients with type 1 diabetes. Diabetes Care.

[CR270] Meijer JW, van Sonderen E, Blaauwwiekel EE, Smit AJ, Groothoff JW, Eisma WH, Links TP (2000). Diabetic neuropathy examination: a hierarchical scoring system to diagnose distal polyneuropathy in diabetes. Diabetes Care.

[CR271] Meijer JW, Smit AJ, Sonderen EV, Groothoff JW, Eisma WH, Links TP (2002). Symptom scoring systems to diagnose distal polyneuropathy in diabetes: the Diabetic Neuropathy Symptom score. Diabet Med.

[CR272] Meijer JW (2003). Clinical diagnosis of diabetic polyneuropathy with the diabetic neuropathy symptom and diabetic neuropathy examination scores. Diabetes Care.

[CR273] Meijer JW, Smit AJ, Lefrandt JD, van der Hoeven JH, Hoogenberg K, Links TP (2005). Back to basics in diagnosing diabetic polyneuropathy with the tuning fork!. Diabetes Care.

[CR274] Meng H, Johnston B, Englesakis M, Moulin DE, Bhatia A (2017). Selective cannabinoids for chronic neuropathic pain: a systematic review and meta-analysis. Anesth Analg.

[CR275] Mick G, Baron R, Finnerup NB, Hans G, Kern KU, Brett B, Dworkin RH (2011). What is localized neuropathic pain? A first proposal to characterize and define a widely used term. Pain Manag.

[CR276] Mickle AD, Shepherd AJ, Mohapatra DP (2015). Sensory TRP channels: the key transducers of nociception and pain. Progress Mol Biol Transl Sci.

[CR277] Mickle AD, Shepherd AJ, Mohapatra DP (2016). Nociceptive TRP channels: sensory detectors and transducers in multiple pain pathologies. Pharmaceuticals (Basel, Switzerland).

[CR278] Mika J (2008). Modulation of microglia can attenuate neuropathic pain symptoms and enhance morphine effectiveness. Pharmacol Rep.

[CR279] Milligan ED, Sloane EM, Watkins LR (2008). Glia in pathological pain: a role for fractalkine. J Neuroimmunol.

[CR280] Minett MS, Falk S, Santana-Varela S, Bogdanov YD, Nassar MA, Heegaard AM, Wood JN (2014). Pain without nociceptors? Nav1.7-independent pain mechanisms. Cell Rep.

[CR281] Moghtaderi A, Bakhshipour A, Rashidi H (2006). Validation of Michigan neuropathy screening instrument for diabetic peripheral neuropathy. Clin Neurol Neurosurg.

[CR282] Moisset X, Bouhassira D (2007). Brain imaging of neuropathic pain. NeuroImage.

[CR283] Moore KA, Kohno T, Karchewski LA, Scholz J, Baba H, Woolf CJ (2002). Partial peripheral nerve injury promotes a selective loss of GABAergic inhibition in the superficial dorsal horn of the spinal cord. J Neurosci.

[CR284] Moore RA, Wiffen PJ, Derry S, Toelle T, Rice AS (2014). Gabapentin for chronic neuropathic pain and fibromyalgia in adults. Cochrane Database Syst Rev.

[CR285] Moore RA, Derry S, Aldington D, Cole P, Wiffen PJ (2015). Amitriptyline for neuropathic pain in adults. Cochrane Database Syst Rev.

[CR286] Moulin D (2014). Pharmacological management of chronic neuropathic pain: revised consensus statement from the Canadian Pain Society. Pain Res Manag.

[CR287] Nardelli P, Khan J, Powers R, Cope TC, Rich MM (2013). Reduced motoneuron excitability in a rat model of sepsis. J Neurophysiol.

[CR289] Nawroth PP (2018). The quest for more research on painful diabetic neuropathy. Neuroscience.

[CR290] Nebuchennykh M, Loseth S, Lindal S, Mellgren SI (2009). The value of skin biopsy with recording of intraepidermal nerve fiber density and quantitative sensory testing in the assessment of small fiber involvement in patients with different causes of polyneuropathy. J Neurol.

[CR291] Ni HD (2016). Glial activation in the periaqueductal gray promotes descending facilitation of neuropathic pain through the p38 MAPK signaling pathway. J Neurosci Res.

[CR292] NICE (2013) Clinical guideline: neuropathic pain-pharmacological management. https://guidance.nice.org.uk/CG173. Accessed July 15 2019

[CR293] North RY (2019). Electrophysiological and transcriptomic correlates of neuropathic pain in human dorsal root ganglion neurons. Brain.

[CR294] Nugraha B (2019). The IASP classification of chronic pain for ICD-11: functioning properties of chronic pain. Pain.

[CR296] O'Brien PD, Sakowski SA, Feldman EL (2014). Mouse models of diabetic neuropath. ILAR J.

[CR298] Olaleye D, Perkins BA, Bril V (2001). Evaluation of three screening tests and a risk assessment model for diagnosing peripheral neuropathy in the diabetes clinic. Diabetes Res Clin Pract.

[CR299] Pantalone KM (2018). Effect of glycemic control on the Diabetes Complications Severity Index score and development of complications in people with newly diagnosed type 2 diabetes. J Diabetes.

[CR300] Papanas N, Ziegler D (2014). Efficacy of alpha-lipoic acid in diabetic neuropathy. Expert Opin Pharmacother.

[CR301] Park J, Park HJ (2017). Botulinum toxin for the treatment of neuropathic pain. Toxins.

[CR302] Pascal MMV (2018). DOLORisk: study protocol for a multi-centre observational study to understand the risk factors and determinants of neuropathic pain. Wellcome Open Res.

[CR303] Patel R, Dickenson AH (2016). Neuronal hyperexcitability in the ventral posterior thalamus of neuropathic rats: modality selective effects of pregabalin. J Neurophysiol.

[CR305] Peltier A, Goutman SA, Callaghan BC (2014). Painful diabetic neuropathy. BMJ.

[CR306] Perkins BA, Olaleye D, Zinman B, Bril V (2001). Simple screening tests for peripheral neuropathy in the diabetes clinic. Diabetes Care.

[CR307] Pfau DB (2014). Quantitative sensory testing in the German Research Network on Neuropathic Pain (DFNS): reference data for the trunk and application in patients with chronic postherpetic neuralgia. Pain.

[CR308] Pinzur MS, Slovenkai MP, Trepman E, Shields NN, Ankle S, Diabetes Committee of American Orthopaedic F (2005). Guidelines for diabetic foot care: recommendations endorsed by the Diabetes Committee of the American Orthopaedic Foot and Ankle Society. Foot Ankle Int.

[CR309] Ponirakis G (2014). The diagnostic accuracy of Neuropad for assessing large and small fibre diabetic neuropathy. Diabet Med.

[CR310] Ponirakis G (2016). NerveCheck: an inexpensive quantitative sensory testing device for patients with diabetic neuropathy. Diabetes Res Clin Pract.

[CR311] Pop-Busui R (2013). Impact of glycemic control strategies on the progression of diabetic peripheral neuropathy in the Bypass Angioplasty Revascularization Investigation 2 Diabetes (BARI 2D). Cohort Diabetes care.

[CR312] Pop-Busui R (2017). Diabetic neuropathy: a position statement by the American Diabetes Association. Diabetes Care.

[CR313] Prabodha LBL, Sirisena ND, Dissanayake VHW (2018). Susceptible and prognostic genetic factors associated with diabetic peripheral neuropathy: a comprehensive literature review. Int J Endocrinol.

[CR315] Qaseem A, Wilt TJ, Kansagara D, Horwitch C, Barry MJ, Forciea MA, Clinical Guidelines Committee of the American College of P (2018). Hemoglobin A1c targets for glycemic control with pharmacologic therapy for nonpregnant adults with type 2 diabetes mellitus: a guidance statement update from the American College of Physicians. Ann Intern Med.

[CR316] Quattrini C (2007). Surrogate markers of small fiber damage in human diabetic neuropathy. Diabetes.

[CR317] Quattrini C, Jeziorska M, Boulton AJ, Malik RA (2008). Reduced vascular endothelial growth factor expression and intra-epidermal nerve fiber loss in human diabetic neuropathy. Diabetes Care.

[CR318] Raghavendra V, Tanga F, DeLeo JA (2003). Inhibition of microglial activation attenuates the development but not existing hypersensitivity in a rat model of neuropathy. J Pharmacol Exp Ther.

[CR319] Rahman M, Griffin SJ, Rathmann W, Wareham NJ (2003). How should peripheral neuropathy be assessed in people with diabetes in primary care? A population-based comparison of four measures. Diabet Med.

[CR320] Rahman W, D'Mello R, Dickenson AH (2008). Peripheral nerve injury-induced changes in spinal alpha(2)-adrenoceptor-mediated modulation of mechanically evoked dorsal horn neuronal responses. J Pain.

[CR322] Raputova J (2017). Sensory phenotype and risk factors for painful diabetic neuropathy: a cross-sectional observational study. Pain.

[CR323] Rauck RL, Shaibani A, Biton V, Simpson J, Koch B (2007). Lacosamide in painful diabetic peripheral neuropathy: a phase 2 double-blind placebo-controlled study. Clin J Pain.

[CR324] Ren K, Dubner R (2010). Interactions between the immune and nervous systems in pain. Nat Med.

[CR325] Rice AS, Smith BH, Blyth FM (2016). Pain and the global burden of disease. Pain.

[CR326] Rodriguez-Gutierrez R, Lipska KJ, McCoy RG (2016). Intensive glycemic control in type 2 diabetes mellitus—a balancing act of latent benefit and avoidable harm: a teachable moment. JAMA Intern Med.

[CR327] Rolke R (2006). Quantitative sensory testing in the German Research Network on Neuropathic Pain (DFNS): standardized protocol and reference values. Pain.

[CR328] Roth T, van Seventer R, Murphy TK (2010). The effect of pregabalin on pain-related sleep interference in diabetic peripheral neuropathy or postherpetic neuralgia: a review of nine clinical trials. Curr Med Res Opin.

[CR329] Saarto T, Wiffen PJ (2007). Antidepressants for neuropathic pain. Cochrane Database Syst Rev.

[CR330] Sadosky A, Mardekian J, Parsons B, Hopps M, Bienen EJ, Markman J (2015). Healthcare utilization and costs in diabetes relative to the clinical spectrum of painful diabetic peripheral neuropathy. J Diabetes Complicat.

[CR331] Safarpour Y, Jabbari B (2018). Botulinum toxin treatment of pain syndromes—an evidence based review. Toxicon.

[CR334] Salter MW, Stevens B (2017). Microglia emerge as central players in brain disease. Nat Med.

[CR335] Sanchez-Ramirez GM, Caram-Salas NL, Rocha-Gonzalez HI, Vidal-Cantu GC, Medina-Santillan R, Reyes-Garcia G, Granados-Soto V (2006). Benfotiamine relieves inflammatory and neuropathic pain in rats. Eur J Pharmacol.

[CR336] Sandkuhler J (2007). Understanding LTP in pain pathways. Mol Pain.

[CR337] Sartor CD, Oliveira MD, Campos V, Ferreira J, Sacco ICN (2018). Cross-cultural adaptation and measurement properties of the Brazilian Version of the Michigan Neuropathy Screening Instrument. Braz J Phys Ther.

[CR338] Schafers M, Svensson CI, Sommer C, Sorkin LS (2003). Tumor necrosis factor-alpha induces mechanical allodynia after spinal nerve ligation by activation of p38 MAPK in primary sensory neurons. J Neurosci.

[CR339] Scholz J, Woolf CJ (2007). The neuropathic pain triad: neurons, immune cells and glia. Nat Neurosci.

[CR340] Scholz J (2005). Blocking caspase activity prevents transsynaptic neuronal apoptosis and the loss of inhibition in lamina II of the dorsal horn after peripheral nerve injury. J Neurosci.

[CR342] Scholz J (2019). The IASP classification of chronic pain for ICD-11: chronic neuropathic pain. Pain.

[CR343] Schratzberger P (2001). Reversal of experimental diabetic neuropathy by VEGF gene transfer. J Clin Investig.

[CR344] Schreiber AK, Nones CF, Reis RC, Chichorro JG, Cunha JM (2015). Diabetic neuropathic pain: physiopathology and treatment. World J Diabetes.

[CR345] Schuh-Hofer S, Fischer J, Unterberg A, Treede RD, Ahmadi R (2018). Spinal cord stimulation modulates descending pain inhibition and temporal summation of pricking pain in patients with neuropathic pain. Acta Neurochir.

[CR346] Schwartz S (2015). A pooled analysis evaluating the efficacy and tolerability of tapentadol extended release for chronic, painful diabetic peripheral neuropathy. Clin Drug Investig.

[CR348] Selvarajah D, Awadh M, Gandhi R, Wilkinson ID, Tesfaye S (2018). Alterations in somatomotor network functional connectivity in painful diabetic neuropathy—a resting state functional magnetic resonance imaging study. Diabetes.

[CR349] Selvarajah D, Heiberg-Gibbons F, Wilkinson ID, Gandhi R, Tesfaye S (2018). A magnetic resonance imaging volumetry study of regional brain atrophy in diabetic peripheral neuropathy. Diabetes.

[CR350] Serra J (2012). Microneurographic identification of spontaneous activity in C-nociceptors in neuropathic pain states in humans and rats. Pain.

[CR351] Shen FY (2015). Alleviation of neuropathic pain by regulating T-type calcium channels in rat anterior cingulate cortex. Mol Pain.

[CR353] Shillo P (2017). Nerve and vascular biomarkers in skin biopsies differentiate painful from painless advanced diabetic peripheral neuropathy. Diabetologia.

[CR354] Shillo P (2019). Reduced vitamin D levels in painful diabetic peripheral neuropathy. Diabetic Med J Br Diabetic Assoc.

[CR352] Shillo PR, Selvarajah D, Greig M, Rao GD, Edden RAE, Wilkinson ID, Tesfaye S (2016). Painless diabetic peripheral neuropathy is characterised by reduced thalamic gamma-aminobutyric acid (GABA). Diabetic Med.

[CR355] Shun CT (2004). Skin denervation in type 2 diabetes: correlations with diabetic duration and functional impairments. Brain.

[CR357] Singh VP, Bali A, Singh N, Jaggi AS (2014). Advanced glycation end products and diabetic complications. Korean J Physiol Pharmacol.

[CR358] Sloan G (2018). A new look at painful diabetic neuropathy. Diabetes Res Clin Pract.

[CR359] Smith BH, Torrance N (2012). Epidemiology of neuropathic pain and its impact on quality of life. Curr Pain Headache Rep.

[CR360] Snyder MJ, Gibbs LM, Lindsay TJ (2016). Treating painful diabetic peripheral neuropathy: an update. Am Fam Physician.

[CR361] Sorensen L, Molyneaux L, Yue DK (2006). The relationship among pain, sensory loss, and small nerve fibers in diabetes. Diabetes Care.

[CR362] Sorge RE (2015). Different immune cells mediate mechanical pain hypersensitivity in male and female mice. Nat Neurosci.

[CR364] Spallone V (2017). Might genetics play a role in understanding and treating diabetic polyneuropathy?. Diabetes Metab Res Rev.

[CR363] Spallone V, Morganti R, D'Amato C, Greco C, Cacciotti L, Marfia GA (2012). Validation of DN4 as a screening tool for neuropathic pain in painful diabetic polyneuropathy. Diabet Med.

[CR365] St John Smith E (2018). Advances in understanding nociception and neuropathic pain. J Neurol.

[CR366] Stolar M (2010). Glycemic control and complications in type 2 diabetes mellitus. Am J Med.

[CR367] Stracke H, Gaus W, Achenbach U, Federlin K, Bretzel RG (2008). Benfotiamine in diabetic polyneuropathy (BENDIP): results of a randomised, double blind, placebo-controlled clinical study. Exp Clin Endocrinol Diabetes.

[CR368] Sumitani M, Ueda H, Hozumi J, Inoue R, Kogure T, Yamada Y, Kogure T (2016). Minocycline does not decrease intensity of neuropathic pain intensity, but does improve its affective dimension. J Pain Palliate Care Pharmacother.

[CR369] Sullivan KA, Lentz SI, Roberts JL, Feldman EL (2008). Criteria for creating and assessing mouse models of diabetic neuropathy. Curr Drug Targets.

[CR370] Sun C (2017). IL-17 contributed to the neuropathic pain following peripheral nerve injury by promoting astrocyte proliferation and secretion of proinflammatory cytokines. Mol Med Rep.

[CR371] Tampin B, Briffa NK, Goucke R, Slater H (2013). Identification of neuropathic pain in patients with neck/upper limb pain: application of a grading system and screening tools. Pain.

[CR372] Tan T, Barry P, Reken S, Baker M, Guideline Development G (2010). Pharmacological management of neuropathic pain in non-specialist settings: summary of NICE guidance. BMJ.

[CR373] Tandrup T, Woolf CJ, Coggeshall RE (2000). Delayed loss of small dorsal root ganglion cells after transection of the rat sciatic nerve. J Comp Neurol.

[CR374] Taylor AM, Mehrabani S, Liu S, Taylor AJ, Cahill CM (2017). Topography of microglial activation in sensory- and affect-related brain regions in chronic pain. J Neurosci Res.

[CR375] Tappe-Theodor A, Kuner R (2014). Studying ongoing and spontaneous pain in rodents—challenges and opportunities. Eur J Neurosci.

[CR376] Tesfaye S, Harris ND, Wilson RM, Ward JD (1992). Exercise-induced conduction velocity increment: a marker of impaired peripheral nerve blood flow in diabetic neuropathy. Diabetologia.

[CR377] Tesfaye S (2005). Vascular risk factors and diabetic neuropathy. N Engl J Med.

[CR378] Tesfaye S (2010). Diabetic neuropathies: update on definitions, diagnostic criteria, estimation of severity, and treatments. Diabetes Care.

[CR379] Tesfaye S, Selvarajah D, Gandhi R, Greig M, Shillo P, Fang F, Wilkinson ID (2016). Diabetic peripheral neuropathy may not be as its name suggests: evidence from magnetic resonance imaging. Pain.

[CR380] Thacker MA (2009). CCL2 is a key mediator of microglia activation in neuropathic pain states. Eur J Pain.

[CR381] Themistocleous AC (2016). The Pain in Neuropathy Study (PiNS): a cross-sectional observational study determining the somatosensory phenotype of painful and painless diabetic neuropathy. Pain.

[CR382] Thrainsdottir S (2003). Endoneurial capillary abnormalities presage deterioration of glucose tolerance and accompany peripheral neuropathy in man. Diabetes.

[CR383] Timmerman H (2017). Investigating the validity of the DN4 in a consecutive population of patients with chronic pain. PLoS ONE.

[CR384] Timmerman H (2018). Avoiding Catch-22: validating the PainDETECT in a population of patients with chronic pain. BMC Neurol.

[CR385] Timmerman H, Wilder-Smith OH, Steegers MA, Vissers KC, Wolff AP (2018). The added value of bedside examination and screening QST to improve neuropathic pain identification in patients with chronic pain. J Pain Res.

[CR386] Torrance N, Elliott AM, Lee AJ, Smith BH (2010). Severe chronic pain is associated with increased 10 year mortality. A cohort record linkage study. Eur J Pain.

[CR387] Torrance N, Ferguson JA, Afolabi E, Bennett MI, Serpell MG, Dunn KM, Smith BH (2013). Neuropathic pain in the community: more under-treated than refractory?. Pain.

[CR388] Tracy JA, Dyck PJ (2008). The spectrum of diabetic neuropathies. Phys Med Rehabil Clin N Am.

[CR389] Treede RD (2019). The role of quantitative sensory testing in the prediction of chronic pain. Pain.

[CR390] Treede RD (2008). Neuropathic pain: redefinition and a grading system for clinical and research purposes. Neurology.

[CR391] Treede RD, Rief W, Barke A, Aziz Q, Bennett MI, Benoliel R, Cohen M, Evers S, Finnerup NB, First MB, Giamberardino MA, Kaasa S, Korwisi B, Kosek E, Lavandʼhomme P, Nicholas M, Perrot S, Scholz J, Schug S, Smith BH, Svensson P, Vlaeyen JWS, Wang SJ (2019). Chronic pain as a symptom or a disease: the IASP Classification of Chronic Pain for the International Classification of Diseases (ICD-11). Pain.

[CR392] Trouvin AP, Perrot S, Lloret-Linares C (2017). Efficacy of venlafaxine in neuropathic pain: a narrative review of optimized treatment. Clin Ther.

[CR393] Truini A (2014). Does the epidermal nerve fibre density measured by skin biopsy in patients with peripheral neuropathies correlate with neuropathic pain?. Pain.

[CR394] Truini A (2018). A cross-sectional study investigating frequency and features of definitely diagnosed diabetic painful polyneuropathy. Pain.

[CR396] Tsuda M, Shigemoto-Mogami Y, Koizumi S, Mizokoshi A, Kohsaka S, Salter MW, Inoue K (2003). P2X4 receptors induced in spinal microglia gate tactile allodynia after nerve injury. Nature.

[CR397] Tsuda M, Inoue K, Salter MW (2005). Neuropathic pain and spinal microglia: a big problem from molecules in "small" glia. Trends Neurosci.

[CR398] Tuveson B, Leffler AS, Hansson P (2007). Heterotopic noxious conditioning stimulation (HNCS) reduced the intensity of spontaneous pain, but not of allodynia in painful peripheral neuropathy. Eur J Pain.

[CR399] UKPSD Study Group U (1998). Intensive blood-glucose control with sulphonylureas or insulin compared with conventional treatment and risk of complications in patients with type 2 diabetes (UKPDS 33). UK Prospective Diabetes Study (UKPDS). Group Lancet.

[CR400] United Nations (2019) Revision of world population prospects. https://population.un.org/wpp/. Accessed 12 07 2019

[CR401] Van Acker K (2009). Prevalence and impact on quality of life of peripheral neuropathy with or without neuropathic pain in type 1 and type 2 diabetic patients attending hospital outpatients clinics. Diabetes Metab.

[CR402] van den Born JC, Hammes HP, Greffrath W, van Goor H, Hillebrands JL (2016). Gasotransmitters in vascular complications of diabetes. Diabetes.

[CR403] van Hecke O, Austin SK, Khan RA, Smith BH, Torrance N (2014). Neuropathic pain in the general population: a systematic review of epidemiological studies. Pain.

[CR404] Vanelderen P (2015). Effect of minocycline on lumbar radicular neuropathic pain: a randomized, placebo-controlled, double-blind clinical trial with amitriptyline as a comparator. Anesthesiology.

[CR406] Vinik EJ, Hayes RP, Oglesby A, Bastyr E, Barlow P, Ford-Molvik SL, Vinik AI (2005). The development and validation of the Norfolk QOL-DN, a new measure of patients' perception of the effects of diabetes and diabetic neuropathy. Diabetes Technol Ther.

[CR407] Vinik A, Rosenstock J, Sharma U, Feins K, Hsu C, Merante D, Investigators D-AUUPIS (2014). Efficacy and safety of mirogabalin (DS-5565) for the treatment of diabetic peripheral neuropathic pain: a randomized, double-blind, placebo- and active comparator-controlled, adaptive proof-of-concept phase 2 study. Diabetes Care.

[CR408] Vinik AI (2016). Capsaicin 8% patch repeat treatment plus standard of care (SOC) versus SOC alone in painful diabetic peripheral neuropathy: a randomised, 52-week, open-label, safety study. BMC Neurol.

[CR409] Vlckova-Moravcova E, Bednarik J, Dusek L, Toyka KV, Sommer C (2008). Diagnostic validity of epidermal nerve fiber densities in painful sensory neuropathies. Muscle Nerve.

[CR410] Vo T, Rice AS, Dworkin RH (2009). Non-steroidal anti-inflammatory drugs for neuropathic pain: how do we explain continued widespread use?. Pain.

[CR411] Vollert J (2016). Quantitative sensory testing using DFNS protocol in Europe: an evaluation of heterogeneity across multiple centers in patients with peripheral neuropathic pain and healthy subjects. Pain.

[CR412] Vollert J (2018). Pathophysiological mechanisms of neuropathic pain: comparison of sensory phenotypes in patients and human surrogate pain models. Pain.

[CR413] von Hehn CA, Baron R, Woolf CJ (2012). Deconstructing the neuropathic pain phenotype to reveal neural mechanisms. Neuron.

[CR414] Wall PD, Gutnick M (1974). Ongoing activity in peripheral nerves: the physiology and pharmacology of impulses originating from a neuroma. Exp Neurol.

[CR415] Wallace MS, Marcotte TD, Umlauf A, Gouaux B, Atkinson JH (2015). Efficacy of inhaled cannabis on painful diabetic neuropathy. J Pain.

[CR417] Wang H (2014). Fulranumab for treatment of diabetic peripheral neuropathic pain: a randomized controlled trial. Neurology.

[CR418] Wang ZT, Yu G, Wang HS, Yi SP, Su RB, Gong ZH (2015). Changes in VGLUT2 expression and function in pain-related supraspinal regions correlate with the pathogenesis of neuropathic pain in a mouse spared nerve injury model. Brain Res.

[CR419] Watanabe K (2018). Altered cerebral blood flow in the anterior cingulate cortex is associated with neuropathic pain. J Neurol Neurosurg Psychiatry.

[CR420] Watson JC, Sandroni P (2016). Central neuropathic pain syndromes. Mayo Clin Proc.

[CR421] Watson CP, Moulin D, Watt-Watson J, Gordon A, Eisenhoffer J (2003). Controlled-release oxycodone relieves neuropathic pain: a randomized controlled trial in painful diabetic neuropathy. Pain.

[CR422] Weintrob N, Amitay I, Lilos P, Shalitin S, Lazar L, Josefsberg Z (2007). Bedside neuropathy disability score compared to quantitative sensory testing for measurement of diabetic neuropathy in children, adolescents, and young adults with type 1 diabetes. J Diabetes Complicat.

[CR423] Wiffen PJ, Derry S, Bell RF, Rice AS, Tolle TR, Phillips T, Moore RA (2017). Gabapentin for chronic neuropathic pain in adults. Cochrane Database Syst Rev.

[CR424] Wild S, Roglic G, Green A, Sicree R, King H (2004). Global prevalence of diabetes: estimates for the year 2000 and projections for 2030. Diabetes Care.

[CR425] Wolff RF, Bala MM, Westwood M, Kessels AG, Kleijnen J (2010). 5% lidocaine medicated plaster in painful diabetic peripheral neuropathy (DPN): a systematic review. Swiss Med Wkly.

[CR426] Wood JN, Boorman JP, Okuse K, Baker MD (2004). Voltage-gated sodium channels and pain pathways. J Neurobiol.

[CR429] Woolf CJ, Salter MW (2000). Neuronal plasticity: increasing the gain in pain. Science (New York, NY).

[CR430] Woolf CJ, Shortland P, Coggeshall RE (1992). Peripheral nerve injury triggers central sprouting of myelinated afferents. Nature.

[CR432] World Health Organization (2001) International classification of functioning, disability and health: ICF. World Health Organization, Geneva. ICD 11. https://icd.who.int/browse11/l-m/en. Accessed 15 Aug 2019

[CR433] Wymer JP, Simpson J, Sen D, Bongardt S, Lacosamide SPSG (2009). Efficacy and safety of lacosamide in diabetic neuropathic pain: an 18-week double-blind placebo-controlled trial of fixed-dose regimens. Clin J Pain.

[CR434] Xanthos DN, Sandkuhler J (2014). Neurogenic neuroinflammation: inflammatory CNS reactions in response to neuronal activity. Nat Rev Neurosci.

[CR435] Xiong Q, Lu B, Ye H, Wu X, Zhang T, Li Y (2015). The diagnostic value of neuropathy symptom and change score, neuropathy impairment score and michigan neuropathy screening instrument for diabetic peripheral neuropathy. Eur Neurol.

[CR436] Yagihashi S, Mizukami H, Sugimoto K (2011). Mechanism of diabetic neuropathy: where are we now and where to go?. J Diabetes Investig.

[CR437] Yang XD, Fang PF, Xiang DX, Yang YY (2019). Topical treatments for diabetic neuropathic pain. Exp Ther Med.

[CR438] Yarnitsky D, Granot M, Nahman-Averbuch H, Khamaisi M, Granovsky Y (2012). Conditioned pain modulation predicts duloxetine efficacy in painful diabetic neuropathy. Pain.

[CR439] Yuan RY, Sheu JJ, Yu JM, Chen WT, Tseng IJ, Chang HH, Hu CJ (2009). Botulinum toxin for diabetic neuropathic pain: a randomized double-blind crossover trial. Neurology.

[CR440] Zamponi GW, Lewis RJ, Todorovic SM, Arneric SP, Snutch TP (2009). Role of voltage-gated calcium channels in ascending pain pathways. Brain Res Rev.

[CR441] Zeilhofer HU, Wildner H, Yevenes GE (2012). Fast synaptic inhibition in spinal sensory processing and pain control. Physiol Rev.

[CR442] Zelman DC, Gore M, Dukes E, Tai KS, Brandenburg N (2005). Validation of a modified version of the Brief Pain Inventory for painful diabetic peripheral neuropathy. J Vasc Nurs.

[CR443] Zhang J, Mense S, Treede RD, Hoheisel U (2017). Prevention and reversal of latent sensitization of dorsal horn neurons by glial blockers in a model of low back pain in male rats. J Neurophysiol.

[CR444] Zhou M, Chen N, He L, Yang M, Zhu C, Wu F (2017). Oxcarbazepine for neuropathic pain. Cochrane Database Syst Rev.

[CR445] Zhuang ZY, Kawasaki Y, Tan PH, Wen YR, Huang J, Ji RR (2007). Role of the CX3CR1/p38 MAPK pathway in spinal microglia for the development of neuropathic pain following nerve injury-induced cleavage of fractalkine. Brain Behav Immun.

[CR446] Ziegler D (2006). Oral treatment with alpha-lipoic acid improves symptomatic diabetic polyneuropathy: the SYDNEY 2 trial. Diabetes Care.

[CR448] Ziegler D, Movsesyan L, Mankovsky B, Gurieva I, Abylaiuly Z, Strokov I (2009). Treatment of symptomatic polyneuropathy with actovegin in type 2 diabetic patients. Diabetes Care.

[CR449] Ziegler D, Keller J, Maier C, Pannek J, German Diabetes A (2014). Diabetic neuropathy. Exp Clin Endocrinol Diabetes.

[CR450] Ziegler D, Landgraf R, Lobmann R, Reiners K, Rett K, Schnell O, Strom A (2018). Painful and painless neuropathies are distinct and largely undiagnosed entities in subjects participating in an educational initiative (PROTECT study). Diabetes Res Clin Pract.

[CR452] Zin CS, Nissen LM, Smith MT, O'Callaghan JP, Moore BJ (2008). An update on the pharmacological management of post-herpetic neuralgia and painful diabetic neuropathy. CNS Drugs.

[CR453] Zorina-Lichtenwalter K, Parisien M, Diatchenko L (2018). Genetic studies of human neuropathic pain conditions: a review. Pain.

[CR454] Zur E (2014). Topical treatment of neuropathic pain using compounded medications. Clin J Pain.

